# Advancing understanding of actinide(iii) (Ac, Am, Cm) aqueous complexation chemistry†

**DOI:** 10.1039/d1sc00233c

**Published:** 2021-03-17

**Authors:** Zachary R. Jones, Maksim Y. Livshits, Frankie D. White, Elodie Dalodière, Maryline G. Ferrier, Laura M. Lilley, Karah E. Knope, Stosh A. Kozimor, Veronika Mocko, Brian L. Scott, Benjamin W. Stein, Jennifer N. Wacker, David H. Woen

**Affiliations:** Los Alamos National Laboratory (LANL) P.O. Box 1663, Los Alamos New Mexico 87545 USA stosh@lanl.gov bstein@lanl.gov; Department of Chemistry, Georgetown University 37th and O Streets NW Washington D.C. 20057 USA

## Abstract

The positive impact of having access to well-defined starting materials for applied actinide technologies – and for technologies based on other elements – cannot be overstated. Of numerous relevant 5f-element starting materials, those in complexing aqueous media find widespread use. Consider acetic acid/acetate buffered solutions as an example. These solutions provide entry into diverse technologies, from small-scale production of actinide metal to preparing radiolabeled chelates for medical applications. However, like so many aqueous solutions that contain actinides and complexing agents, 5f-element speciation in acetic acid/acetate cocktails is poorly defined. Herein, we address this problem and characterize Ac^3+^ and Cm^3+^ speciation as a function of increasing acetic acid/acetate concentrations (0.1 to 15 M, pH = 5.5). Results obtained *via* X-ray absorption and optical spectroscopy show the aquo ion dominated in dilute acetic acid/acetate solutions (0.1 M). Increasing acetic acid/acetate concentrations to 15 M increased complexation and revealed divergent reactivity between early and late actinides. A neutral Ac(H_2_O)_6__(1)_(O_2_CMe)_3__(1)_ compound was the major species in solution for the large Ac^3+^. In contrast, smaller Cm^3+^ preferred forming an anion. There were approximately four bound O_2_CMe^1−^ ligands and one to two inner sphere H_2_O ligands. The conclusion that increasing acetic acid/acetate concentrations increased acetate complexation was corroborated by characterizing (NH_4_)_2_M(O_2_CMe)_5_ (M = Eu^3+^, Am^3+^ and Cm^3+^) using single crystal X-ray diffraction and optical spectroscopy (absorption, emission, excitation, and excited state lifetime measurements).

## Abstract 2

Actinide complexation from aqueous acetic acid/acetate buffered solutions is described. The number of water ligands was directly correlated with the acetate concentration and characterized by X-ray absorption and optical spectroscopy.

## Graphical abstract

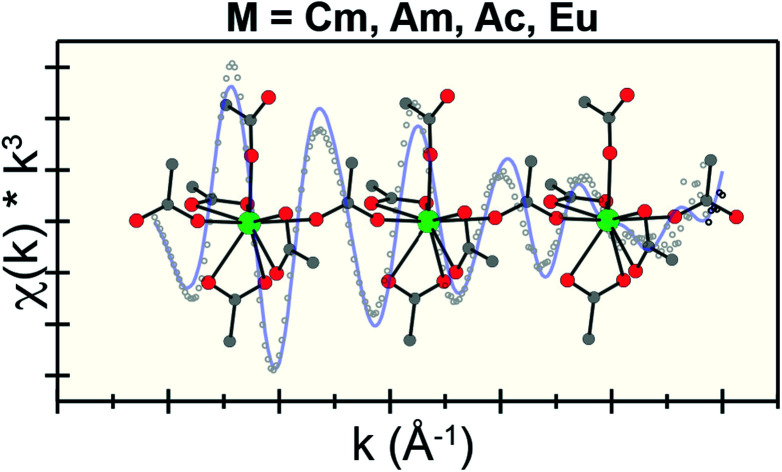

## Introduction

Advancing understanding of actinides in aqueous media could have a widespread impact on solving relevant problems in actinide science. Almost all aspects of technologically relevant actinide chemistry rely – at some point – on aqueous actinide processing. The impact spans from large-scale plutonium metal production to actinide environmental monitoring, and attempting to achieve energy security using nuclear power.^1^ Unfortunately, speciation and reactivity of actinides in aqueous solutions is often poorly defined. One of many examples includes actinides dissolved in buffered ammonium acetate and acetic acid solutions, (NH_4_)O_2_CMe_(aq)_:HO_2_CMe_(aq)_. Beyond a few focused studies,^2–9^ this stock solution is poorly characterized because of the inherent challenges associated with handling highly radioactive actinides. Interpreting spectroscopic results from actinides in this complicated aqueous environment is also challenging in comparison to well-defined organic solutions as well as when frozen in the solid-state.^10,11^ Nevertheless, the reality of this intellectual void is still surprising given that actinide-containing (NH_4_)O_2_CMe_(aq)_:HO_2_CMe_(aq)_ solutions provide convenient entry into small-scale production of actinide metals,^12–14^ as starting materials to label chelators with alpha-emitting radionuclides for therapeutic applications,^15^ in aqueous synthetic efforts,^16,17^ and (to a more limited extent) for actinide separations.^18^ From this perspective, there is a clear need to better define and control actinide speciation within (NH_4_)O_2_CMe_(aq)_:HO_2_CMe_(aq)_ buffered stock solutions.

Having poorly characterized starting materials, like actinide (NH_4_)O_2_CMe_(aq)_:HO_2_CMe_(aq)_ stock solutions, is not specific to the field of aqueous 5f-element chemistry. It is, instead, a systemic problem. Use of poorly defined starting materials transcends many aspects of materials science, biology, organic, and inorganic chemistry, *etc.* Consider numerous reports published in journals focused on characterizing “simple” starting materials, those that need to be better understood because of widespread use.^19–23^ These modest contributions are highly cited and foundational. From that perspective, we drew analogy to actinide (NH_4_)O_2_CMe_(aq)_:HO_2_CMe_(aq)_ buffered stock solutions and embarked to change the current state of affairs. Additional motivation came from operational needs at Los Alamos National Laboratory (LANL) to improve actinide chelation technologies and establish a small-scale capability for actinide metal production.

To achieve our goal and advance understanding of actinide speciation in buffered (NH_4_)O_2_CMe_(aq)_:HO_2_CMe_(aq)_ (pH = 5.5) stock solution starting materials, we carefully selected aqueous environments where (NH_4_)O_2_CMe_(aq)_:HO_2_CMe_(aq)_ was present at three different concentrations. Particular attention was paid to maintaining relevance, in that we wanted our results to be directly applicable (or easily extrapolated) to the above-mentioned application space. First, actinides in solutions with dilute (NH_4_)O_2_CMe_(aq)_:HO_2_CMe_(aq)_ (0.1 M) concentrations were interrogated. Here, the An^3+^ and O_2_CMe^1−^ ions were solutes in an aqueous matrix. Second, actinides in concentrated (NH_4_)O_2_CMe_(aq)_:HO_2_CMe_(aq)_ (15 M) solutions, where water was now a solute rather than a solvent, were studied. Third, we characterized actinides in a solution with an intermediate (NH_4_)O_2_CMe_(aq)_:HO_2_CMe_(aq)_ (4 M) concentration. Under these conditions there was an abundance of the coordinating O_2_CMe^1−^ ions; meanwhile, water also remained in huge excess. A series of complementary characterization methods that were compatible with the complicated sample types (small quantities of highly radioactive actinides in aqueous media) were deployed, namely solution-phase X-ray absorption spectroscopy (XAS) and solution-phase and solid-state optical spectroscopy (absorption, emission, excitation, and excited state lifetime measurements). Results were evaluated within the context of structural data from single crystals grown from acetate solutions. Of particular relevance was characterization of Am(O_2_CMe)_5_^2−^, Cm(O_2_CMe)_5_^2−^, and Eu(O_2_CMe)_5_^2−^ using single crystal X-ray diffraction.

## Results and discussion

### Synthesis of bisammonium metal(iii) pentakisacetate, (NH_4_)_2_M(O_2_CMe)_5_, M = Eu^3+^, Am^3+^, Cm^3+^

Both solution and solid-state 5f-element syntheses relied on preparing chemically pure stock solutions of Ac^3+^_(aq)_, Am^3+^_(aq)_, and Cm^3+^_(aq)_ (see Methods section for details). From these nitrate stock solutions, syntheses for bisammonium metal(iii) pentakisacetate, (NH_4_)_2_M(O_2_CMe)_5_, involved first precipitating hydrated f-element hydroxides, M(OH)_3_·*x*H_2_O with ammonium hydroxide, NH_4_OH_(aq)_ (14.5 M). Granted, this hydroxide drop was only attempted with actinide (Cm^3+^, Am^3+^) and lanthanide (Eu^3+^) elements that could be handled on the macroscopic scale (mg quantities), not Ac^3+^. The resulting residue was washed with water – to remove residual nitrate – and dissolved in an aqueous solution of ammonium acetate, (NH_4_)O_2_CMe_(aq)_ (10 M). Slow evaporation of these solutions at room temperature for 2.5 weeks routinely produced single crystals of (NH_4_)_2_M(O_2_CMe)_5_ that were suitable for single crystal X-ray diffraction. We estimated that the crystalline yields from Cm^3+^ and Am^3+^ were similar to that from Eu^3+^ at ∼26%. Given the good success with Cm^3+^, Am^3+^, and Eu^3+^ attempts to extend the procedure to trans-curium elements were made. Applying this procedure to Cf^3+^ has unfortunately not yet produced single crystals that could be characterized by X-ray diffraction.

### Structure of bisammonium metal(iii) pentakisacetate, (NH_4_)_2_M(O_2_CMe)_5_, M = Eu^3+^, Am^3+^, Cm^3+^

**Fig. 1:**
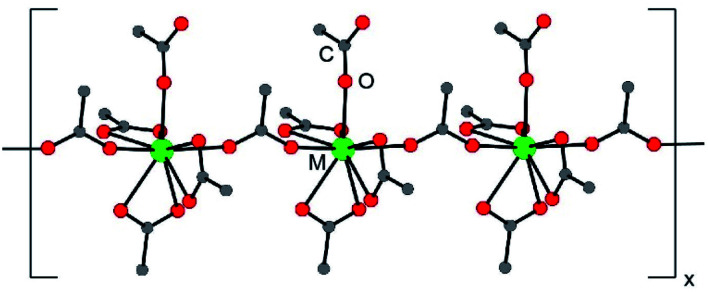
A ball and stick representation of the (NH_4_)_2_M(O_2_CMe)_5_ (M^3+^ = Eu, Am, Cm) structures obtained by single crystal X-ray diffraction. The NH_4_^1+^ cations have been omitted.

The (NH_4_)_2_M(O_2_CMe)_5_ compounds were isomorphous and crystallized in the monoclinic, *P*2_1_/*n* space group as one-dimensional chains (Fig. 1). Each structure contained five acetate ligands bound to M^3+^ cations. Three acetates were bidentate (κ^2^) and terminal, one was monodentate and terminal (κ^1^), and one was monodentate and bridging (µ^1^:κ^1^). From the perspective of the metal, local geometries were best described as having a pseudo-pentagonal plane of five oxygen atoms, capped on one side by a single oxygen atom and on the other side by three oxygen atoms. All nine of these oxygens were associated with acetate ligands. Hence, the compounds were homoleptic and no bound H_2_O was observed. This 9-coordinate geometry approached a mono-capped square antiprism,^24^ which is well-established for lanthanides^25^ and actinides.^26^ Average metal–O_O2CMe_ distances have been provided in Table 1. These acetate structures constitute rare examples of minor actinides characterized by single crystal X-ray diffraction.^27–30^

**Table 1:** Average M–O_***O***_2_CMe_ (Å) bond distances determined by single crystal X-ray diffraction measurements made on (NH_4_)_2_M(O_2_CMe)_5_ (M = Eu, Am, Cm). Uncertainty is reported as the standard deviation from the mean at 1*σ*^a^

Compound	M–O_***O***_2_CMe_ (Å)
(NH_4_)_2_Eu(O_2_CMe)_5_	2.46 ± 0.09
(NH_4_)_2_Am(O_2_CMe)_5_	2.50 ± 0.08
(NH_4_)_2_Cm(O_2_CMe)_5_	2.49 ± 0.08

a The standard deviation is high due to the mixture of (κ^1^)- *vs.* (κ^2^)-coordination environments.

### Solution-phase speciation

To determine if the solid-state structures described above were maintained in solution, An^3+^ L_3_-edge X-ray absorption near edge spectroscopy (XANES), An^3+^ L_3_-edge extended X-ray absorption fine structure (EXAFS), ultraviolet-visible spectroscopy (UV-vis), and time resolved excited state fluorescence lifetime (TRFL) measurements were carried out on the M^3+^ cations dissolved in acetate solutions. Scope for XAS experiments was confined to two actinide size extremes; the largest +3 5f-element (Ac^3+^, ionic radius = 1.12 Å for a coordination number of 6) was compared against one of the smallest actinides (Cm^3+^, ionic radius = 0.97 Å for a coordination number of 6). Another constraint was being able to obtain reasonably-sized quantities of these radionuclides (µg for Ac and mg for Cm).^31^ Actinide L_3_-edge XAS spectra were evaluated as a function of increasing concentrations of (NH_4_)O_2_CMe_(aq)_:HO_2_CMe_(aq)_ buffer; 0.2 and 15 M for Ac^3+^ and 0.1, 4, and 15 M for Cm^3+^ at a constant pH of 5.5. Notice that the ionic strength changed substantially through this series of (NH_4_)O_2_CMe_(aq)_:HO_2_CMe_(aq)_ matrixes. Interpretations for the Cm^3+^ L_3_-edge EXAFS results – particularly regarding the number of H_2_O ligands bound to Cm^3+^ – were validated by optical measurements (UV-vis and TRFL). These data were comparatively evaluated against Eu^3+^, because (just as in the Cm^3+^ case) UV-vis and TRFL spectroscopies are well-established methods for determining Eu^3+^ hydration numbers.^32,33^ To most effectively communicate results to the reader, the An^3+^ L_3_-edge XANES measurements (An = Ac, Cm) are discussed first. Then, the EXAFS results are discussed; starting with dilute (NH_4_)O_2_CMe_(aq)_:HO_2_CMe_(aq)_ buffered solutions followed by EXAFS measurements made in concentrated (NH_4_)O_2_CMe_(aq)_:HO_2_CMe_(aq)_ buffered solutions. We conclude the analyses by describing combined use of Cm^3+^ L_3_-edge EXAFS spectroscopy with Cm^3+^ and Eu^3+^ UV-vis and TRFL spectroscopy in intermediate (NH_4_)O_2_CMe_(aq)_:HO_2_CMe_(aq)_ (4 M) buffered solutions.

### An^3+^ L_3_-edge XANES measurements in (NH_4_)O_2_CMe_(aq)_:HO_2_CMe_(aq)_ buffered solutions

**Fig. 2:**
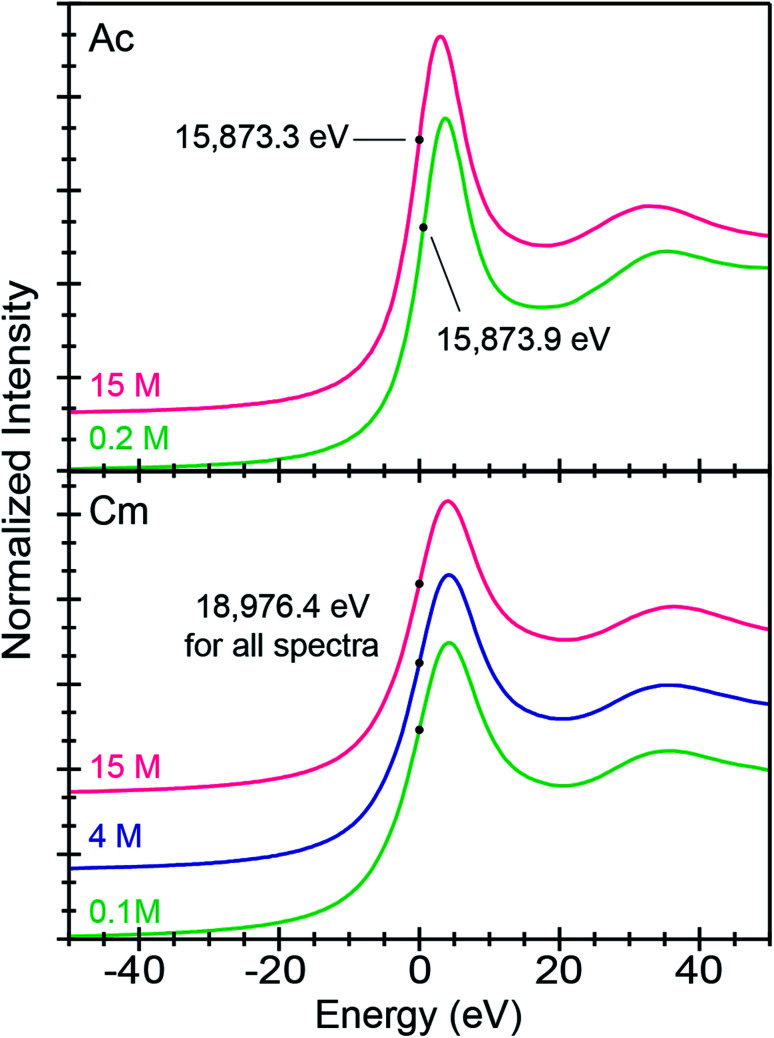
The background subtracted and normalized room temperature solution-phase An^3+^ L_3_-edge XANES spectra from Ac^3+^ (top) dissolved in (NH_4_)O_2_CMe_(aq)_:HO_2_CMe_(aq)_ (15 M and 0.2 M, pH = 5.5) solutions and Cm^3+^ (bottom) dissolved in (NH_4_)O_2_CMe_(aq)_:HO_2_CMe_(aq)_ (15 M, 4 M, 0.1 M, pH = 5.5) solutions. Spectra are displayed with a slight *y*-offset for clarity. The dots show the inflection points.

All background subtracted and normalized An^3+^ L_3_-edge XANES spectra were dominated by an absorption peak superimposed on an absorption edge (Fig. 2). These absorption peaks primarily resulted from electric-dipole allowed actinide 2p-electronic excitations to final states that contained 6d-character. Using Ac^3+^ as an example, this involved transitions between the 2p^6^ … 5f^0^ 6d^0^ ground-state and 2p^5^ … 5f^0^ 6d^1^ excited-state electronic configurations. The inflection point for Ac^3+^ in dilute (NH_4_)O_2_CMe_(aq)_:HO_2_CMe_(aq)_ (0.2 M) at 15 873.9(1) eV was higher by 0.6 eV from the 15 873.3(1) eV recorded in concentrated (NH_4_)O_2_CMe_(aq)_:HO_2_CMe_(aq)_ (15 M). An even greater difference was observed between Ac^3+^ in (NH_4_)O_2_CMe_(aq)_:HO_2_CMe_(aq)_ (15 M) and the Ac^3+^-aquo ion [15 874.3(1) eV], reported previously (Table 2).^34–36^ This 1 eV edge energy difference may be related to substantial electronic structure changes that accompany An–O_2_CMe bond formation. Notice, with an estimated uncertainty of 0.1 eV, inflection points from the Ac^3+^-aquo ion were equivalent to that from the Ac^3+^ spectrum in dilute (NH_4_)O_2_CMe_(aq)_:HO_2_CMe_(aq)_ (0.2 M) at 1*σ*, but easily discernable from the inflection point observed in concentrated (NH_4_)O_2_CMe_(aq)_:HO_2_CMe_(aq)_ (15 M).

**Table 2:** Inflection points and peak position for the room temperature An^3+^ L_3_-edge XANES spectra from Ac^3+^ and Cm^3+^ cations dissolved in varied concentrations of (NH_4_)O_2_CMe_(aq)_:HO_2_CMe_(aq)_ (pH = 5.5)^a^

Analyte (concentration)	Inflection point (eV)	1st peak position (eV)	2nd peak position (eV)
Ac (0.2 M)	15 873.9(1)	15 876.8(1)	15 908.1(1)
Ac (15 M)	15 873.3(1)	15 876.3(1)	15 906.3(1)
Cm (0.1 M)	18 976.4(1)	18 980.8(1)	19 011.7(1)
Cm (4 M)	18 976.4(1)	18 980.7(1)	19 011.6(1)
Cm (15 M)	18 976.4(1)	18 980.6(1)	19 012.8(1)

a Inflection point and the peak positions were defined graphically where the second and first derivative of the data equaled zero, respectively.

Similar observations were not made for Cm^3+^. Instead, Cm^3+^ L_3_-edge inflection points from all three (NH_4_)O_2_CMe_(aq)_:HO_2_CMe_(aq)_ solutions (0.1, 4, and 15 M) were equivalent. These 18 976.4(1) eV inflection point values were also identical to that determined previously for the Cm^3+^ aqua ion in HNO_3_ (0.05 M) at 18 976.4(1) eV.^34^ At this stage, it is unclear why the Ac^3+^ inflection points were more sensitive to ligand environments than Cm^3+^; although, it is tempting to propose that these appreciable Ac^3+^ L_3_-edge inflection point shifts resulted from more substantial orbital mixing in Ac–ligand bonds *vs.* the Cm–ligand bonds.

### Speciation in dilute (NH_4_)O_2_CMe_(aq)_:HO_2_CMe_(aq)_ buffered solutions (0.2 and 0.1 M)

**Fig. 3:**
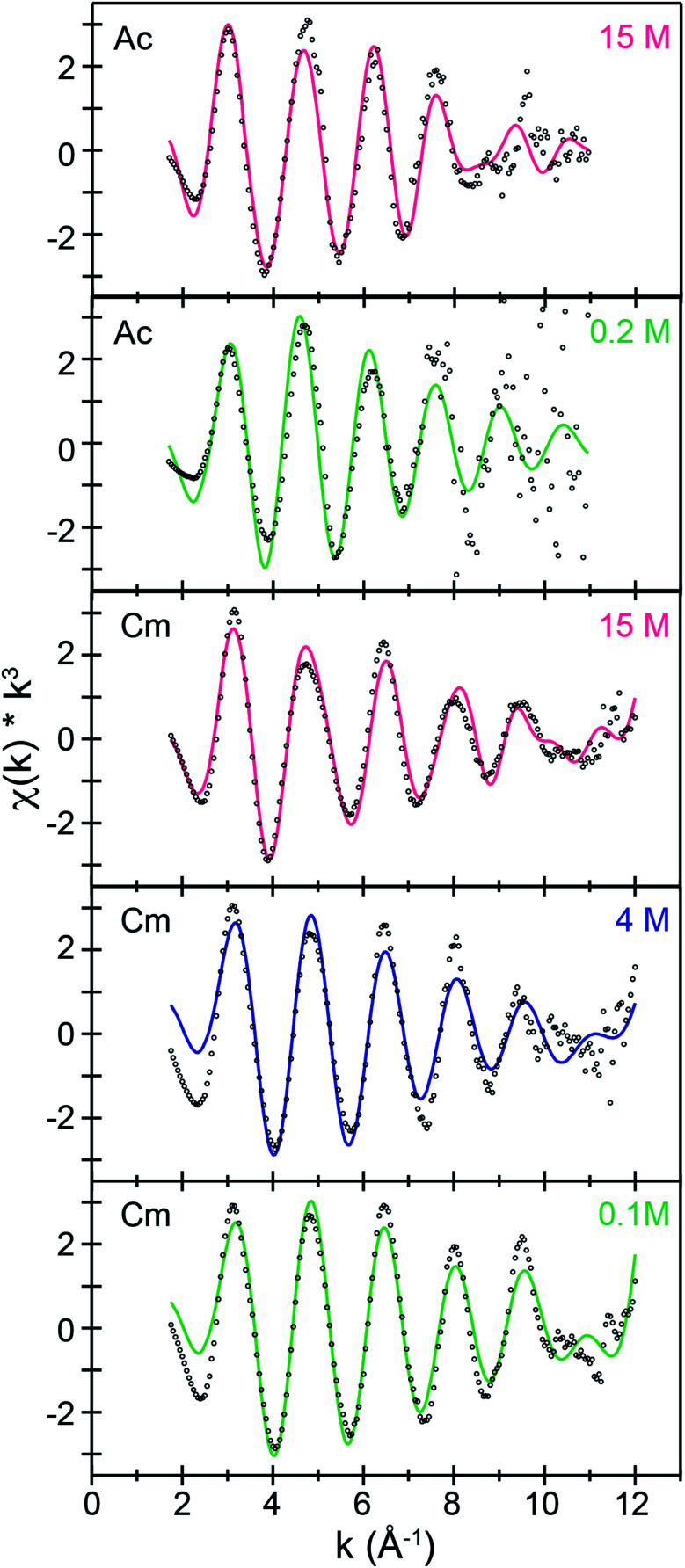
*k* ^3^-Weighted room temperature Ac^3+^ and Cm^3+^ L_3_-edge EXAFS spectra displaying the *χ*(*k*) function (points) with non-linear curve fitting (lines) from Ac^3+^ dissolved in (NH_4_)O_2_CMe_(aq)_:HO_2_CMe_(aq)_ (15 M and 0.2 M, pH = 5.5) solutions and from Cm^3+^ dissolved in (NH_4_)O_2_CMe_(aq)_:HO_2_CMe_(aq)_ (15 M, 4 M, 0.1 M, pH = 5.5) solutions.

**Fig. 4:**
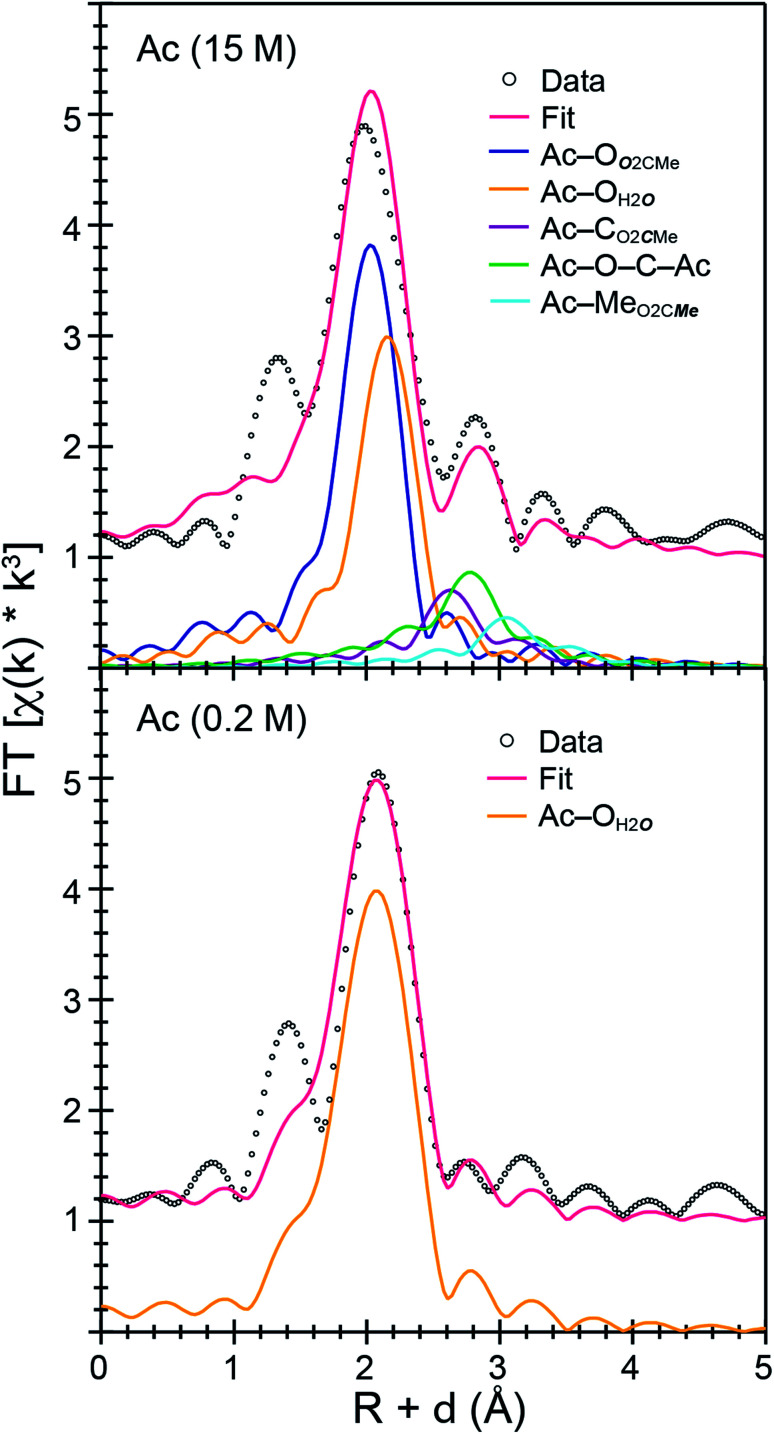
Fourier transform of room temperature Ac^3+^ L_3_-edge EXAFS spectra displaying the magnitude and imaginary space (points) with non-linear curve fitting (lines) of Ac^3+^ cations dissolved in (NH_4_)O_2_CMe_(aq)_:HO_2_CMe_(aq)_ (top, 15 M) and (bottom, 0.2 M), pH = 5.5.

**Fig. 5:**
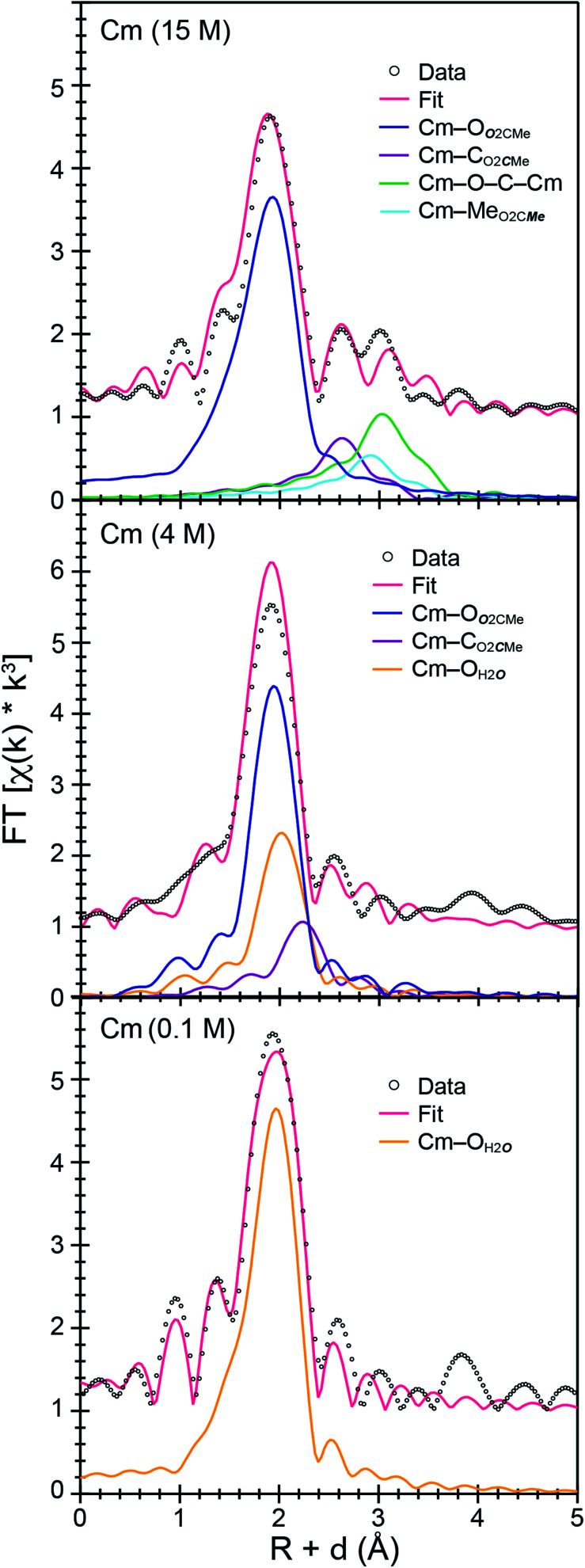
Fourier transform of room temperature Cm^3+^ L_3_-edge EXAFS spectra displaying the magnitude and imaginary space (points) with non-linear curve fitting (lines) of Cm^3+^ cations dissolved in (NH_4_)O_2_CMe_(aq)_:HO_2_CMe_(aq)_ (top, 15 M), (middle, 4 M), and (bottom, 0.1 M), buffered pH = 5.5.

Room temperature *k*^3^-weighted An^3+^ L_3_-edge EXAFS spectra and Fourier transformed data from Cm^3+^ and Ac^3+^ have been compared in Fig. 3–5 as a function of (NH_4_)O_2_CMe_(aq)_:HO_2_CMe_(aq)_ buffered concentration. Models of the data were constrained to maintain a reasonable number of free variables and avoided overparameterization of the fits.^37^ Endpoint energies varied from 9.5 (Ac^3+^) to 10.5 (Cm^3+^) Å^−^^1^ in *k*-space, which restricted the EXAFS resolution to 0.17 and 0.15 Å for Ac^3+^ and Cm^3+^. The limited resolution was a product of collecting the data at room temperature and having only small quantities of the actinide analyte. The *k*^3^-weighted An^3+^ L_3_-edge EXAFS measurements from Ac^3+^ and Cm^3+^ in dilute (NH_4_)O_2_CMe_(aq)_:HO_2_CMe_(aq)_ buffered solutions (0.2 M for Ac^3+^ and 0.1 M for Cm^3+^) were best described as single-phase wave functions. In both cases there was only one intense peak in the Fourier transform of the *k*^3^-weighted data near 2 (Å, *δ* + *R*). Spectra from dilute (NH_4_)O_2_CMe_(aq)_:HO_2_CMe_(aq)_ buffered solutions were modeled as homoleptic aquo cations, An(H_2_O)_*x*_^3+^, with single Ac–O_H2***O***_ and Cm–O_H2***O***_ scattering pathways. The amplitude reduction factors (*S*_0_^2^) were fixed at 0.9 and the mean-squared displacement variables (*σ*^2^), actinide–H_2_O distances (An–O_H2***O***_), and H_2_O coordination numbers (*N*_H2O_) were allowed to converge to reasonable values (Table 3 and Fig. 5). For Ac^3+^, the EXAFS data suggested that the first coordination-sphere contained 9(1) waters at a long Ac–O_H2***O***_ distance of 2.64(1) Å. For Cm^3+^, the fit suggested 8(1) water molecules with a substantially shorter Cm–O_H2***O***_ distance of 2.47(1) Å, consistent with the smaller Cm^3+^ ionic radius.

Structural metrics from the Ac^3+^ spectra in dilute (NH_4_)O_2_CMe_(aq)_:HO_2_CMe_(aq)_ buffered solutions (0.2 M) were equivalent to the two other Ac^3+^-aquo reports, namely Ac^3+^ L_3_-edge EXAFS studies in dilute triflic and dilute nitric acid.^34^ All three of these Ac^3+^-aquo data sets also agreed (within uncertainty) to predictions from molecular dynamics simulations on Ac(H_2_O)_*x*_^3+^. There was agreement with the original predictions we published using *ab initio* molecular dynamics (AIMD) calculations. That level of theory predicted 9 H_2_O ligands with an average Ac–O_H2***O***_ distance of 2.69 ± 0.11 Å (8 ps time interval).^35^ There was also agreement with EXAFS simulations by Marcos and co-Workers using classical molecular dynamics simulations (Ac–O_H2***O***_ 2.66 ± 0.02 Å and a coordination number ranging from 8 to 9 over a 10 ns time interval).^38^ Our results agreed serendipitously with previous XANES simulations.^36,38^ However, those XANES calculations focused on an artifact associated with a monochromator glitch in the previously published Ac^3+^-aquo measurement made in dilute triflic acid solutions. That glitch occurred when the Si 110 crystal used at the Stanford Synchrotron Radiation Lightsource's (SSRL's) Beam Line 11-2 end-station was oriented at *Φ* = 0° and the line shape was not affiliated with the sample (Fig. S1 and S2†). We are excited to have now provided two examples of “glitch” free Ac^3+^-aquo L_3_-edge XANES spectra (with the Si 110 crystal oriented at *Φ* = 90°), the data reported here in dilute (NH_4_)O_2_CMe_(aq)_:HO_2_CMe_(aq)_ and that reported previously in ref. 32. Full data sets for the two Ac^3+^-aquo ions (as well other spectra associated with this study) have been provided in the ESI.†

The solution phase speciation for Cm^3+^ in dilute buffered solutions (0.1 M) was in agreement with previous studies suggesting that the Cm^3+^-aquo ion existed in dilute acetate solutions.^9^ Data herein is also comparable with previous reports on the Cm^3+^-aquo ion. For instance, the coordination numbers and bond-distances that we obtained were consistent with single crystal data from Cm(H_2_O)_9_^3+^,^39,40^ which showed six H_2_O ligands with short Cm–O_H2***O***_ distances [2.453(1) Å] and three H_2_O ligands with longer Cm–O_H2***O***_ distances, 2.545(1) Å. Our data also agreed with previous reports that modeled the Cm^3+^-aquo ion in solution.^41^ An early report from Allen and co-Workers found 10.2(3) water molecules with a Cm–O_H2***O***_ distance of 2.450(2) Å.^40^ Subsequent efforts confirmed Allen's results and expanded upon what was understood regarding the Cm^3+^ hydration environment. For instance, Skanthakumar and co-Workers published high-energy X-ray scattering (HEXS) measurements showing 8.8(3) H_2_O ligands at 2.48(1) Å.^39^ They also contributed EXAFS data (higher resolution data than that from Allen) that revealed two H_2_O shells, an inner shell of H_2_O ligands at 2.450(2) Å followed closely by a second shell of H_2_O ligands at 2.63(2) Å; coordination numbers were fixed at six and three, respectively.^39,40^ Our measurement included here [8(1) waters, Cm–O_H2***O***_ = 2.47(1) Å], which admittedly had lower resolution than that from Skanthakumar, was consistent with all four of the reports. It also agreed with the Cm^3+^-aquo EXAFS study we published previously in dilute HNO_3_ solutions [0.05 M; 9.6(7) H_2_O; Cm–O_H2***O***_ = 2.47(1) Å].^34^

### Speciation in concentrated (NH_4_)O_2_CMe_(aq)_:HO_2_CMe_(aq)_ buffered solutions (15 M)

**Scheme 1:**
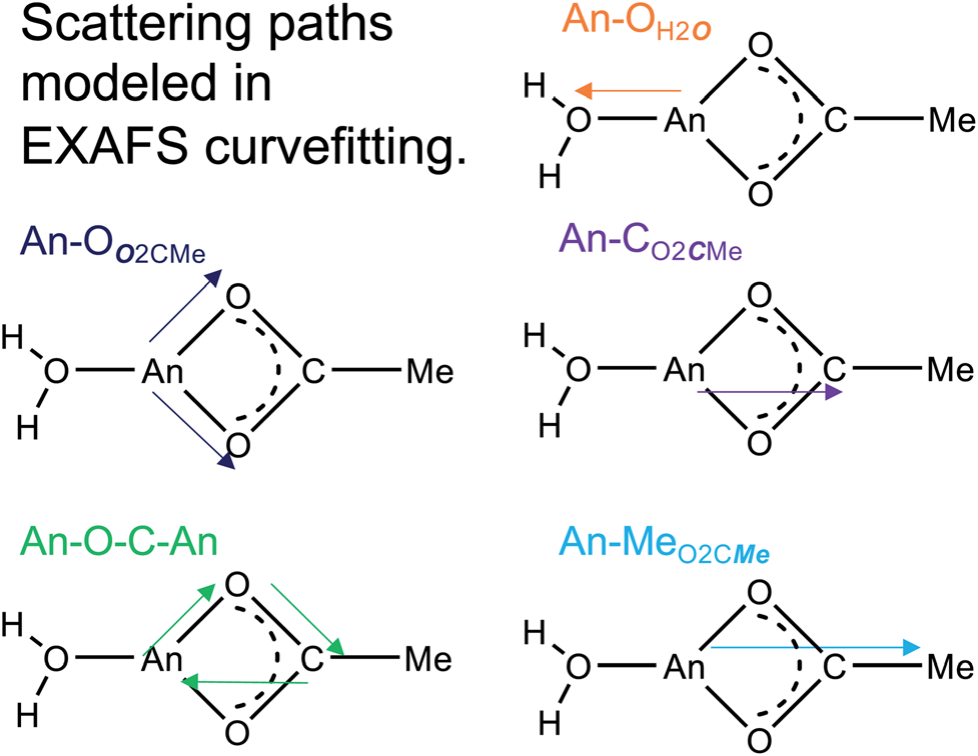
Simulated scattering paths used to model the Ac^3+^ and Cm^3+^ L_3_-edge EXAFS data. Single scattering and directly bound O (yellow or blue) and C (purple) were modeled for the first shell. The higher-order features were modeled with a triangle multiple scattering path (green), and a linear scattering path from the methyl backbone (light blue).

The Ac^3+^ and Cm^3+^ L_3_-edge EXAFS results in concentrated (NH_4_)O_2_CMe_(aq)_:HO_2_CMe_(aq)_ (15 M) buffered solutions were distinct from dilute buffered solutions (0.2 and 0.1 M). Most notably, concentrated solutions did not generate spectra that contained single-phase wavefunctions. Instead, multiple contributions associated with actinide-bound O_2_CMe^1−^ were present. In the Ac^3+^ case, there were two well-resolved and short Ac–O scattering pathways in the innermost coordination sphere. One was modeled with 6(1) oxygen atoms from neutral H_2_O ligands with an Ac–O_H2***O***_ distance of 2.56(6) Å (Table 3). This shell was followed closely by 5(2) oxygen atoms from O_2_CMe^1−^ monoanions with an Ac–O_***O***2CMe_ distance of 2.69(6) Å. Higher frequency contributions were modeled with the 3(1) C_O2***C***Me_ atoms, 3(1) Me_O2C***Me***_ atoms, and 6(1) four-component Ac–O–C–Ac multiple scattering pathways at 3.19(3), 3.72(3), and 3.41(3) Å, respectively (Scheme 1). The EXAFS spectra showed an average stoichiometry of Ac(H_2_O)_6(1)_(O_2_CMe)_3(1)_ (acetate values based on the C_O2***C***Me_ coordination number).

**Table 3:** Parameters used to model the An^3+^ (An = Ac and Cm) L_3_-edge EXAFS spectra from Ac^3+^ and Cm^3+^ dissolved in (NH_4_)O_2_CMe_(aq)_:HO_2_CMe_(aq)_ buffered (pH 5.5) aqueous solutions. Note, the amplitude reduction factors (*S*_0_^2^) were fixed at 0.9

Analyte (concentration)	Path	*R* (Å)	CN	*σ* ^2^×10^3^
Ac (0.2 M)^a^	Ac–O_H_2_***O***_	2.64(1)	9(1)	12(3)
Ac (15 M)^a^	Ac–O_H_2_***O***_	2.56(6)	6(1)	9(3)^b^
	Ac–O_***O***_2_CMe_	2.69(6)	5(2)	9(3)^b^
	Ac–C_O_2_***C***Me_	3.19(3)	3(1)^c^	4(2)^d^
	Ac–C_O_2_C***Me***_	3.72(3)	3(1)^c^	4(2)^d^
	Ac–O–C–Ac	3.41(3)	6(1)	4(2)^d^
Cm (0.1 M)^e^	Cm–O_H_2_***O***_	2.47(1)	8(1)	10(2)
Cm (4 M)^e^	Cm–O_***O***_2_CMe+H_2_***O***_	2.47(2)	9(1)	12(2)
	Cm–C_O_2_***C***Me_	2.91(1)	2(1)	3(1)
Cm (15 M)^e^	Cm–O_***O***_2_CMe+H_2_***O***_	2.46(1)	9.1(8)	12(5)
	Cm–C_O_2_***C***Me_	3.27(4)^f^	4.5(5)^g^	7(4)^h^
	Cm–C_O_2_C***Me***_	3.56(4)^f^	4.5(5)^g^	7(4)^h^
	Cm–O–C–Cm	3.71(3)	9.1(8)	7(4)^h^

a Ac samples: *S*_o_^2^ (fixed) = 0.9, Δ*E*_o_ = 9.0(3) eV 1.15 ≤ *R* ≤ 4 Å, 2.3 ≤ *k* ≤ 11.0, *N*_ind_ = 15.8.

b Constrained equivalently.

c Constrained equivalently.

d Constrained equivalently.

e Cm samples: *S*_o_^2^ (fixed) = 0.9, Δ*E*_o_ = −0.3(5) eV 1.0 ≤ *R* ≤ 3.5 Å, 1.4 ≤ *k* ≤ 12.0, *N*_ind_ = 16.9.

f Constrained to the same shift (*Δ*) in *R* (Å).

g Constrained equivalently.

h Constrained equivalently.

It is interesting to note that the short 2.56(6) Å Ac–O distance refined as the Ac–O_H2***O***_ distance and not the Ac–O_***O***2CMe_. Initially, this concerned us, as we expected the anionic O_2_CMe^1−^ ligand would have shorter Ac–O distances. However, the literature contains many single crystal X-ray diffraction reports for other f-elements that show neutral H_2_O ligands bound at shorter distances than anionic ligands, *e.g.* NO_3_^1−^ to Th^4+^, U^4+^, Np^4+^, Pu^3+^, Am^3+^, Nd^3+^, Sm^3+^, Gd^3+^, and Eu^3+^.^20,42–48^ This structural data was consistent with the EXAFS model included here. It is unclear to us why metal–ligand bonds would be shorter for neutral H_2_O *vs.* anionic O_2_CMe^1−^ ligands. However, we also acknowledge that at room temperature and in solution rapid O_2_CMe^1−^ and H_2_O exchange seemed possible.

For Cm^3+^, the low frequency contributions refined as a single shell of 9.1(8) oxygen atoms with a Cm–O distance of 2.46(1) Å. Longer-range electron scattering was modeled with 4.5(5) C_O_2_***C***Me_ atoms at 3.27(4) Å, followed by 4.5 Me_O_2_C***Me***_ (constrained to the same value as the Cm–C_O_2_***C***Me_ group) at 3.56(4) Å, and 9.1(8) Cm–C–O–Cm four coordinate multiple scattering pathways at 3.71(3) Å. These Cm–O_2_CMe distances were consistent with the single crystal X-ray diffraction data in (NH_4_)_2_Cm(O_2_CMe)_5_ (*vide supra*), *e.g.* single crystal Cm–O_***O***_2_CMe_ distances ranged 2.348(3) to 2.558(3) Å and averaged 2.49 ± 0.08 Å (uncertainty reported as the standard deviation from the mean at 1*σ*). Overall, these data suggested that the Cm^3+^ inner coordination sphere was dominated by O_2_CMe^1−^ in concentrated (NH_4_)O_2_CMe_(aq)_:HO_2_CMe_(aq)_ (15 M) buffer. Evaluating these EXAFS data alongside the time-resolved fluorescence lifetime (TRFL) measurements described below led us to conclude that the Cm^3+^ cation had approximately four bound O_2_CMe^1−^ ligands and one to two inner sphere H_2_O ligands. Contributions from bound H_2_O to the Cm^3+^ L_3_-edge EXAFS spectrum were small in comparison to Ac^3+^. These results highlight appreciable differences in speciation between Ac^3+^ and Cm^3+^. For example, the smaller and more Lewis acidic Cm^3+^ cation attracted primarily anionic O_2_CMe^1−^ ligands (fewer bound H_2_O). On the other hand, the inner coordination sphere for the larger and less Lewis acidic Ac^3+^ cation had more neutral H_2_O ligands than O_2_CMe^1−^ anions.

Efforts to include monodentate κ^1^-O_2_CMe^1−^ binding in the Ac^3+^ and Cm^3+^ L_3_-edge EXAFS spectra for concentrated (NH_4_)O_2_CMe_(aq)_:HO_2_CMe_(aq)_ (15 M) buffered solutions converged to reasonable values, but did not appreciably improve the fits. The κ^1^-O_2_CMe^1−^ scattering pathway only stole intensity from the bidentate (κ^2^-O_2_CMe^1−^) ligand. Unfortunately, we did not believe that we could reliably quantify the number of κ^1^-bound *vs.* κ^2^-chelated O_2_CMe^1−^ groups. The quality of the Cm^3+^ and Ac^3+^ L_3_-edge EXAFS data was insufficient to evaluate κ^1^- *vs.* κ^2^-O_2_CMe^1−^ binding while remaining below the Nyquist limit of free variables (∼75% of the maximum number of variables allowed for the fitted region). Without additional data to inform the model, and given that hydration and ligand interconversion (κ^2^-O_2_CMe^1−^ to κ^1^-O_2_CMe^1−^) are dynamic processes in the solution phase (especially at room temperature), we acknowledge the likelihood of κ^1^-bound O_2_CMe^1−^ and refrain from overinterpreting this EXAFS data by commenting on acetate monodenticity.

### Speciation in intermediate (NH_4_)O_2_CMe_(aq)_:HO_2_CMe_(aq)_ buffered solutions (4 M)

The Cm^3+^ L_3_-edge EXAFS spectrum from intermediate (NH_4_)O_2_CMe_(aq)_:HO_2_CMe_(aq)_ buffered (4 M) solutions was similar (but not equivalent) to that described above in dilute acetate. Subtracting the Cm^3+^ spectrum obtained in 0.1 M (NH_4_)O_2_CMe_(aq)_:HO_2_CMe_(aq)_ from the 4 M data (in energy, *k* space) quantified this similarity. This residual analysis gave a reasonably flat line at zero; average deviation from zero was 13% between 1.4 and 12.0 Å^−1^. It is likely no coincidence that fitting the data as a linear combination of the 15 and 0.1 M (NH_4_)O_2_CMe_(aq)_:HO_2_CMe_(aq)_ spectra showed that the Cm^3+^ speciation favored the 15 M extreme also by only 13%. This would account for around 0.6 coordinated O_2_CMe^1−^ ligands. Shell-by-shell fitting that included bound O_2_CMe^1−^ and H_2_O showed an inner shell of 9(1) oxygen atoms with a Cm–O distance of 2.47(2) Å. There was also a second shell with 2(1) carbon atoms at 2.91(1) Å. Contributions to the EXAFS spectrum from the methyl group of the acetate ligand were too weak to detect, likely owing to a combination of the small number of coordinated O_2_CMe^1−^ ligands and to the long distance from Cm^3+^ to the outer methyl substituent. Magnitudes and uncertainties associated with coordination numbers, bond distances, and Debye–Waller factors (*σ*^2^) for the Cm–O_H2***O***_, Cm–O_***O***2CMe_, and Cm–C_O2***C***Me_ scattering pathways – as well as for the ionization energy displacement variable (*E*^0^) – refined reasonably well, and the number of independent variables (9) associated with adding the O_2_CMe^1−^ ligand were well within an acceptable limit. This heteroleptic model was also intuitively reasonable, as we assumed that increasing the amount of O_2_CMe^1−^ from 0.2 M to 4 M would increase complexation of Cm^3+^ by O_2_CMe^1−^. However, shell-by-shell fitting of the data as a homoleptic Cm^3+^-aquo ion (only H_2_O ligands and no O_2_CMe^1−^) provided an equivalently good model. This fit showed a single shell with 9(1) H_2_O ligands with a Cm–O_H2***O***_ distance of 2.47(2) Å. Hence, additional data was sought to inform the model and determine if Cm^3+^ was bound by O_2_CMe^1−^ in (NH_4_)O_2_CMe_(aq)_:HO_2_CMe_(aq)_ buffered (4 M) solutions or if the Cm^3+^-aquo ion persisted in this matrix.

**Fig. 6:**
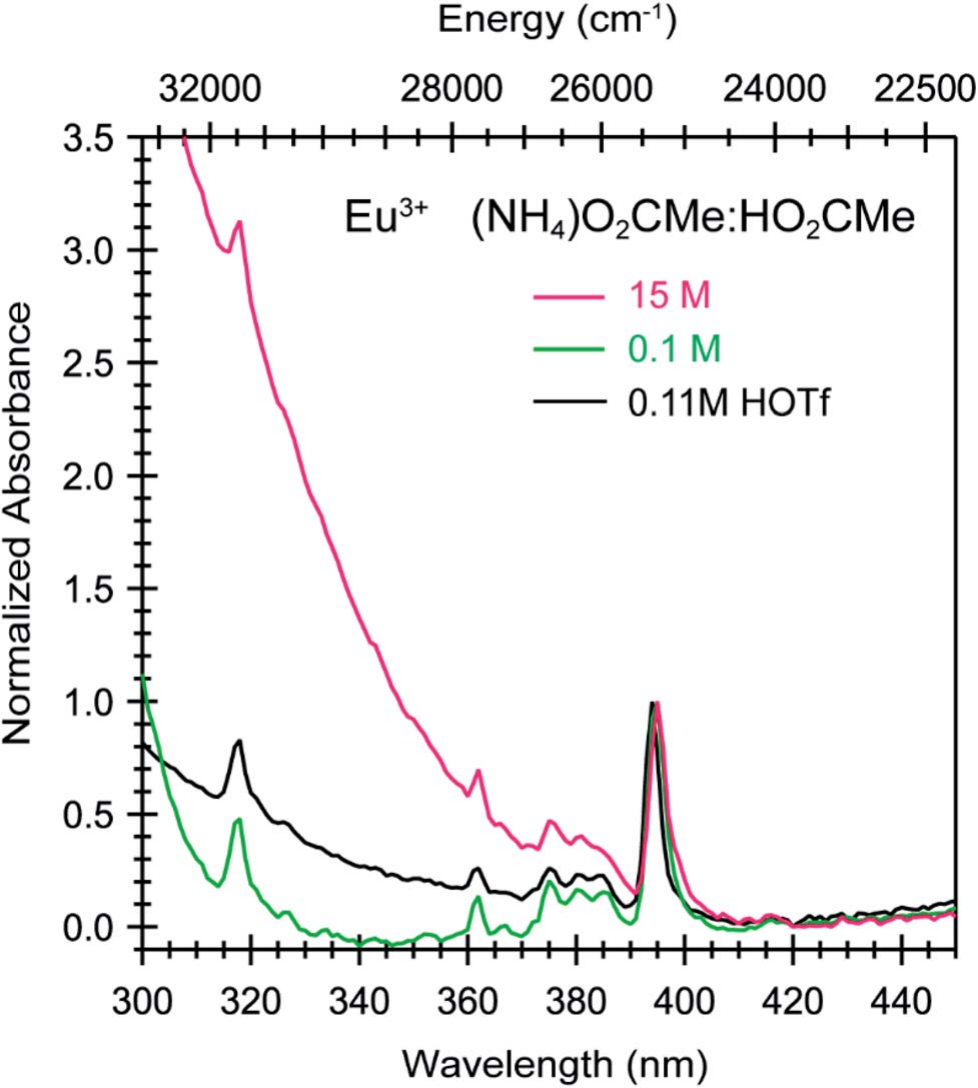
Absorption spectra from room temperature solutions containing Eu^3+^ dissolved in HOTf_(aq)_ (0.11 M, black trace) and (NH_4_)O_2_CMe_(aq)_:HO_2_CMe_(aq)_ (pH = 5.5) buffered solutions (0.1 M green trace; 15 M pink trace).

**Fig. 7:**
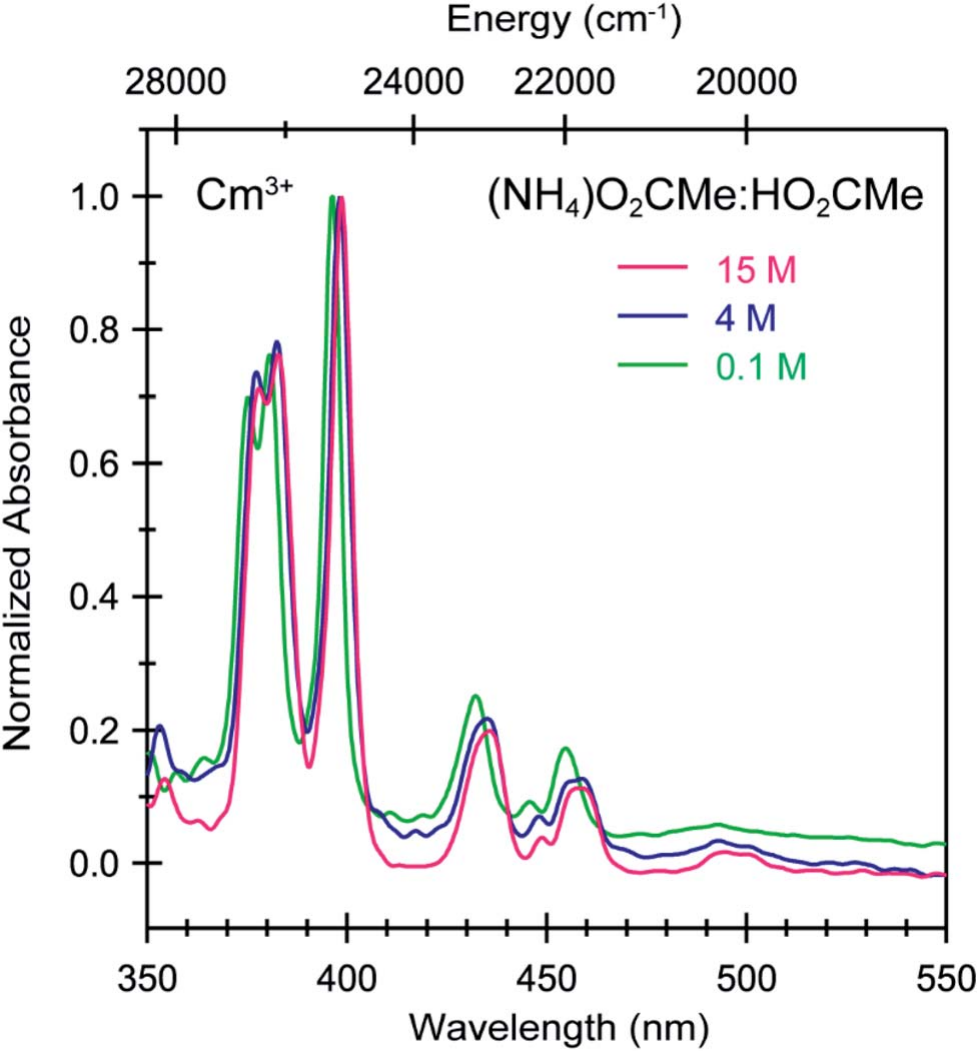
Absorbance spectra from room temperature solutions containing Cm^3+^ dissolved in (NH_4_)O_2_CMe_(aq)_:HO_2_CMe_(aq)_ buffered solutions (0.1 M green trace, 4 M blue trace, 15 M pink trace, pH = 5.5).

**Fig. 8:**
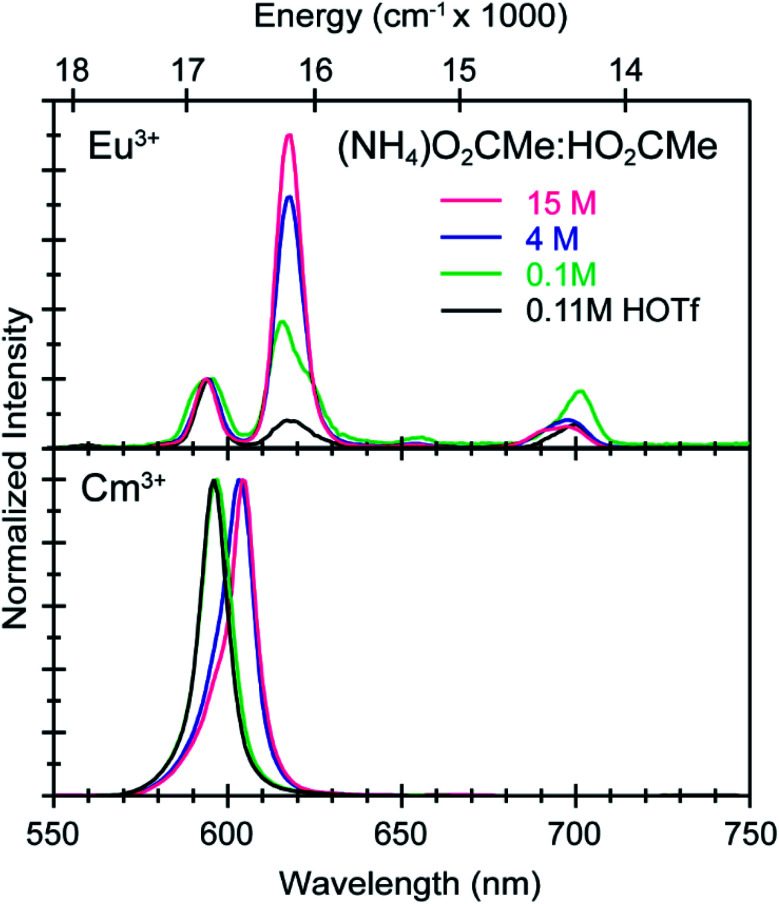
Emission spectra from room temperature solutions containing Eu^3+^ (top) and Cm^3+^ (bottom) dissolved in HOTf_(aq)_ (0.11 M black trace) and (NH_4_)O_2_CMe_(aq)_:HO_2_CMe_(aq)_ buffered solutions (0.1 M green trace, 4 M blue trace, 15 M pink trace, pH = 5.5).

**Fig. 9:**
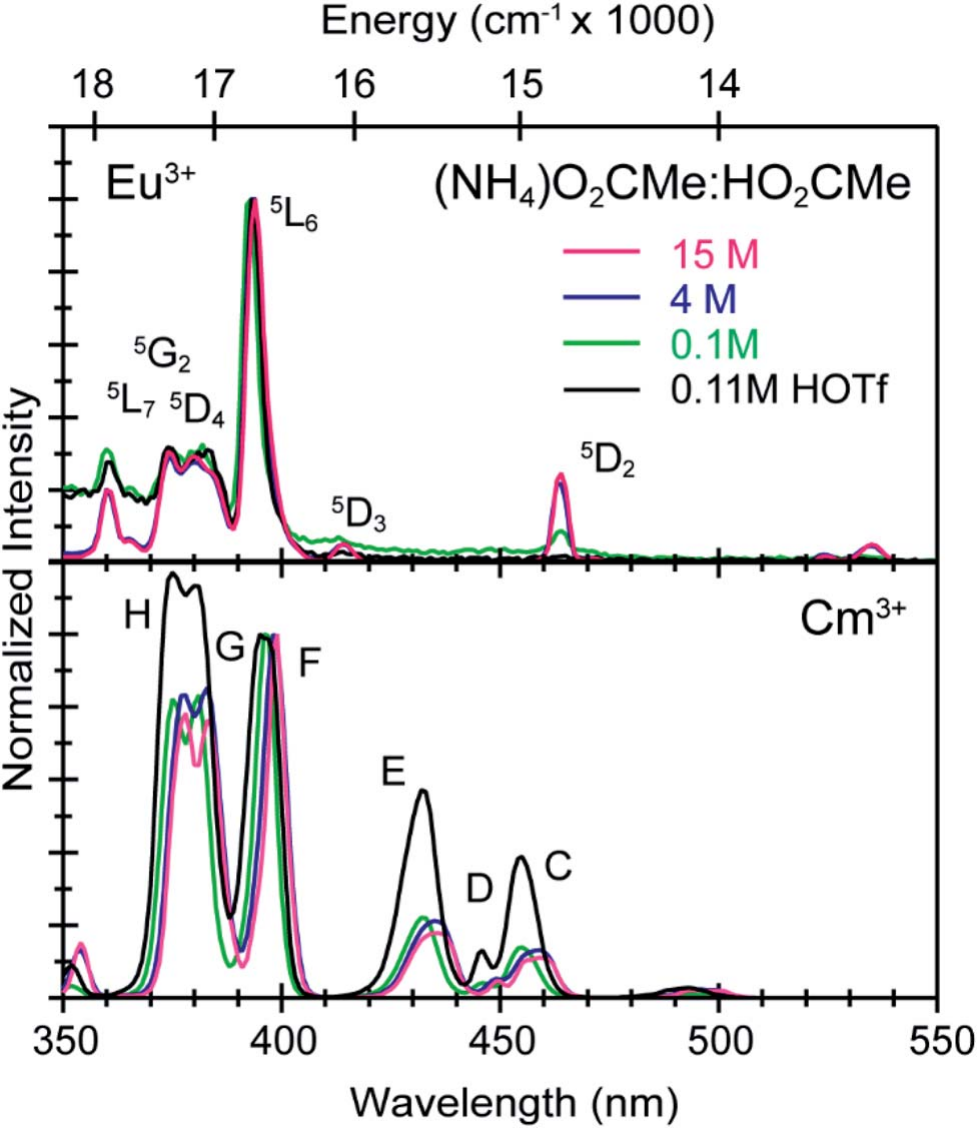
Excitation spectra from room temperature solutions containing Eu^3+^ (top) and Cm^3+^ (bottom) dissolved in HOTf_(aq)_ (0.11 M, black trace) and (NH_4_)O_2_CMe_(aq)_:HO_2_CMe_(aq)_ buffered solutions (0.1 M green trace, 4 M blue trace, 15 M pink trace, pH = 5.5).

**Fig. 10:**
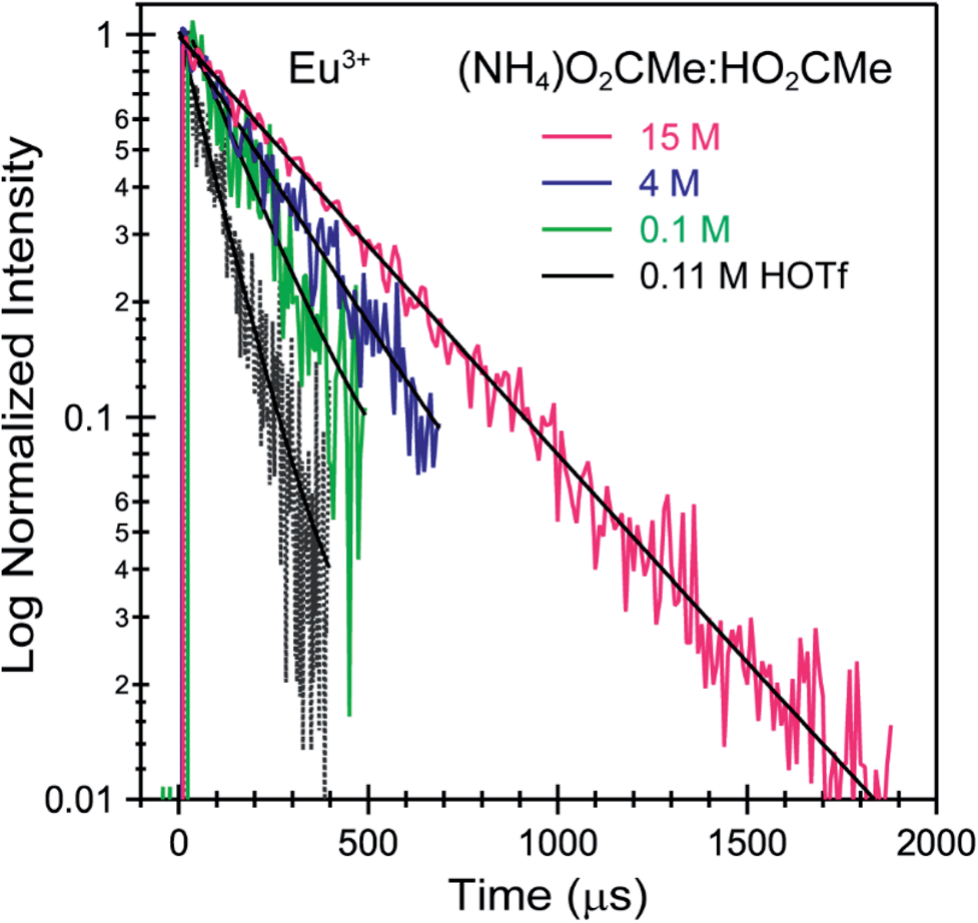
TRFL spectra from room temperature solutions containing Eu^3+^ dissolved in HOTf_(aq)_ (0.11 M black trace) and (NH_4_)O_2_CMe_(aq)_:HO_2_CMe_(aq)_ buffered solutions (0.1 M green trace, 4 M blue trace, 15 M pink trace, pH = 5.5). Fits to the data are shown as solid black traces. The excitation energy was fixed at 25 320 cm^−1^.

**Fig. 11:**
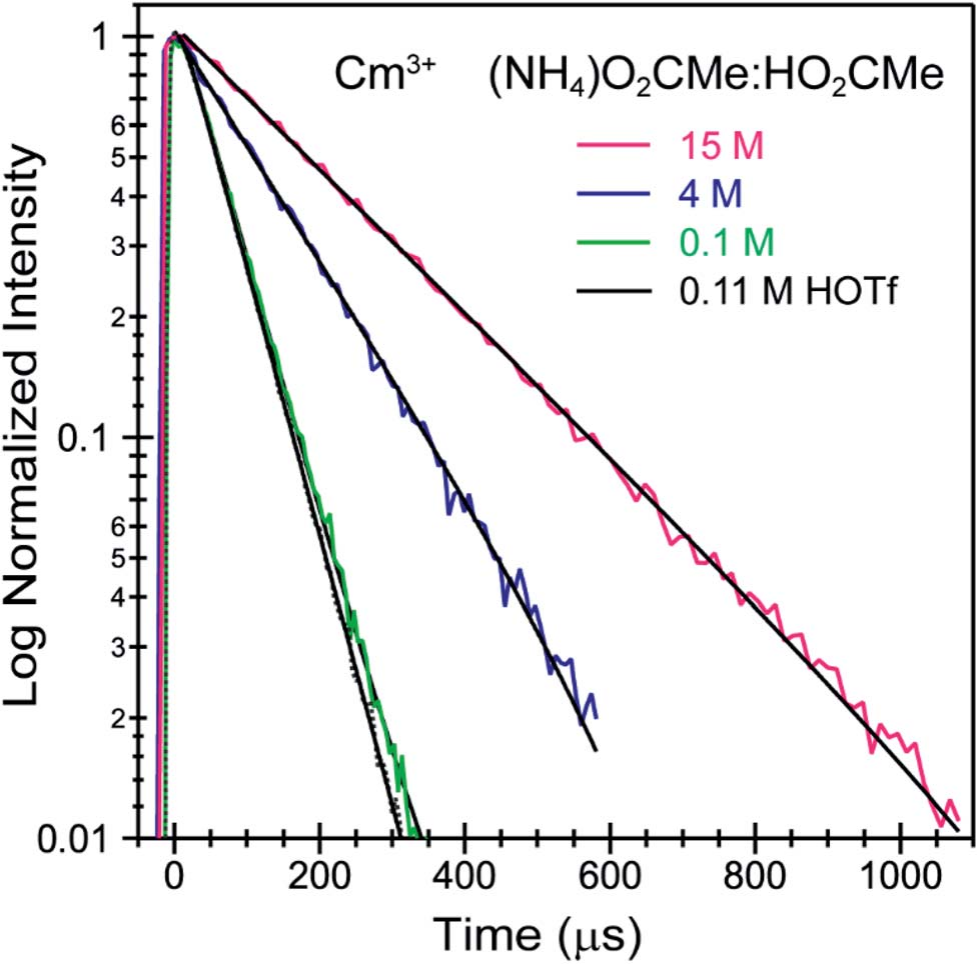
TRFL spectra from room temperature solutions containing Cm^3+^ dissolved in HOTf_(aq)_ (0.11 M black trace) and (NH_4_)O_2_CMe_(aq)_:HO_2_CMe_(aq)_ buffered solutions (0.1 M green trace, 4 M blue trace, 15 M pink trace, pH = 5.5). Fits to the data are shown as solid black traces. The excitation energy was fixed at 25 450 cm^−1^.

To better constrain the EXAFS models in terms of metal bound H_2_O *vs.* O_2_CMe^1−^ in intermediate (NH_4_)O_2_CMe_(aq)_:HO_2_CMe_(aq)_ buffered solutions (4 M), efforts were made to qualitatively identify acetate complexation using UV-vis (Fig. 6 and 7), emission (Fig. 8), and excitation (Fig. 9) spectroscopy. We anticipated that displacement of H_2_O by O_2_CMe^1−^ would alter the intensities for many features associated within the excitation spectra, as H_2_O binding provides non-radiative decay pathways for the optically accessed excited state species that are inaccessible in complexes containing only O_2_CMe^1−^ ligands. We also expected that substituting a stronger field and bidentate O_2_CMe^1−^ ligand for the weaker field and monodentate H_2_O ligand would increase the ligand field splitting and reduce the symmetry of the resulting acetate complexes. Taken together, these changes should systematically shift the energy for the emission peaks as a function of (NH_4_)O_2_CMe_(aq)_:HO_2_CMe_(aq)_ concentration relative to the Cm^3+^-aquo ion; albeit, the exact impact of these substitutions on the intensity and energy can be difficult to predict. The UV-vis absorption spectra were anticipated to exhibit systematic changes, as well, upon increased O_2_CMe^1−^ complexation. Conclusions regarding substitution of O_2_CMe^1−^ for H_2_O based on these qualitative excitation and absorption measurements were then quantitatively substantiated using time-resolved fluorescence lifetime (TRFL) spectroscopy (Fig. 10 and 11). In this scenario, the emission lifetimes were expected to increase with increasing (NH_4_)O_2_CMe_(aq)_:HO_2_CMe_(aq)_ concentration if O_2_CMe^1−^ displaced H_2_O because H_2_O provides non-radiative relaxation pathways. Confidence and credibility for interpreting the Cm^3+^ optical measurements were established by comparative analyses with analogous Eu^3+^ measurements, simply because it was easier to handle and study large quantities of non-radioactive and abundant Eu^3+^ over radioactive and rare Cm^3+^.

To calibrate the optical response of Cm^3+^ and Eu^3+^ as a function of (NH_4_)O_2_CMe_(aq)_:HO_2_CMe_(aq)_ concentration, excitation spectra were initially obtained in aqueous environments that were well-established for stabilizing “true” Cm^3+^- and Eu^3+^-aquo ions, namely in dilute HOTf_(aq)_ (0.11 M; Fig. 9). For the Cm^3+^-aquo ion, excitation spectra were collected with an emission wavelength (*λ*_em_) of 598 nm (16 720 cm^−1^). The 5f → 5f transitions were labeled using conventional alphabetic designators; Z → C (454 to 458 nm), Z → D (445–449 nm), Z → E (432 to 436 nm), Z → F (396 to 399 nm), Z → G (380 to 383 nm) and Z → H (375 to 378 nm).^49^ The excitation spectrum (*λ*_em_ = 619 nm, 6170 cm^−1^) from the Eu^3+^-aquo ion also contained 4f → 4f transitions attributed previously to ^7^F_0_ → ^5^D_2_ (465 nm), ^7^F_1_ → ^5^D_3_ (415 nm), ^7^F_0_ → ^5^L_6_ (395 nm), a combination of ^7^F_1_ → ^5^L_7_ and ^7^F_0_→^5^G_2_ (375 to 385), and ^7^F_0_ → ^5^D_4_ (360 nm).^33^ Intensity changes for subsequently obtained Cm^3+^ and Eu^3+^ spectra were monitored after normalizing peak maxima for the Z → F (for Cm^3+^) and ^7^F_0_ → ^5^L_6_ (for Eu^3+^) transitions to unity, at 1.0.

**Scheme 2:**
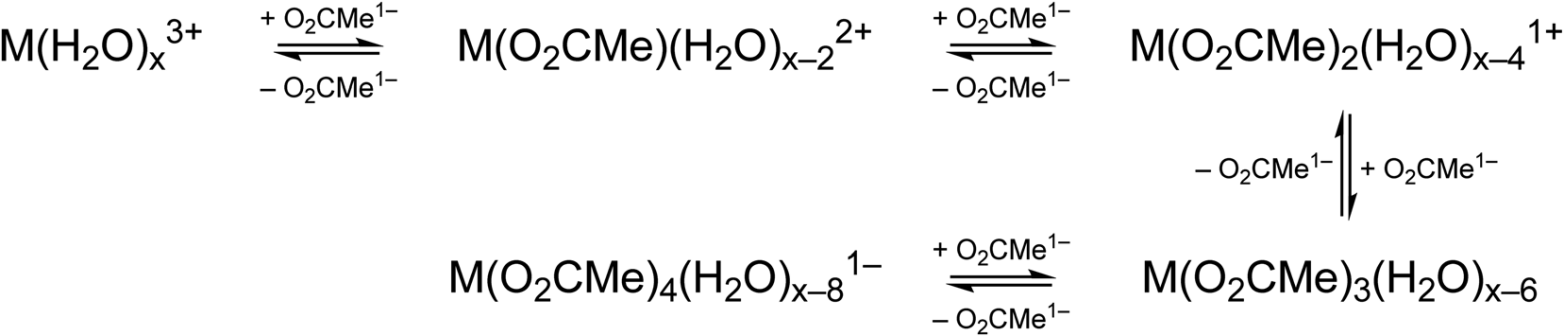
Potential metal speciation in (NH_4_)O_2_CMe_(aq)_:HO_2_CMe_(aq)_ buffered stock solutions.

Changing the matrix from dilute HOTf_(aq)_ (0.11 M) to the dilute (NH_4_)O_2_CMe_(aq)_:HO_2_CMe_(aq)_ buffered solution (0.1 M, pH = 5.5) and then increasing the (NH_4_)O_2_CMe_(aq)_:HO_2_CMe_(aq)_ concentration (to 4 M and 15 M) imparted more noticeable changes on the excitation spectra from Cm^3+^ than Eu^3+^. For Cm^3+^, these changes manifested primarily as shifts for the Z → F, Z → E, and Z → C peaks to lower energy. Additionally, the Z → E and Z → C features decreased in intensity. For Eu^3+^, the intensity for the ^7^F_0_ → ^5^D_2_ transitions increased while the high energy transitions (>25 000 cm^−1^) decreased. Although we were unable to exactly characterize the origin for these intensity changes and energy shifts, it is qualitatively obvious that the excitation spectra reflected how increasing (NH_4_)O_2_CMe_(aq)_:HO_2_CMe_(aq)_ concentration pushed the speciation profile away from the aquo ion and toward complexes with larger numbers of coordinated acetate ligands (Scheme 2). Absorption and emission spectra from Cm^3+^ and Eu^3+^ provided similar qualitative evidence of increasing acetate complexation (Fig. 6–8).

Time resolved fluorescence lifetime (TRFL) measurements were made to quantify hydration numbers for Cm^3+^ and Eu^3+^ as a function of increasing (NH_4_)O_2_CMe_(aq)_:HO_2_CMe_(aq)_ buffered concentrations (Fig. 10 and 11). This approach represents one of the most powerful techniques available for quantifying H_2_O bound by Cm^3+^ and Eu^3+^ in solution. In these experiments, excitation energies were fixed at 393 nm (25 450 cm^−1^) for Cm^3+^ and 395 nm (25 320 cm^−1^) for Eu^3+^. Subsequent analyte emission was monitored at 598 or 605 nm (16 720 or 16 530 cm^−1^ for Cm^3+^) and 619 nm (16 170 cm^−1^ for Eu^3+^), and the emission decay kinetics were measured. Emission decay rates were modeled with a bi-exponential function (e^−*k*_instr_*T*^ + e^−*k*_obs_*T*^). There was initially a short decay rate (*k*_instr_) associated with the instrument response function (IRF, >100 µs) that was followed by a longer decay rate (*k*_obs_) associated with the Cm^3+^ and Eu^3+^ complexes (>100 µs, *k*_obs_ being the analyte lifetime; Tables 4, 5). The number of bound water molecules (*N*_H_2_O_) was determined using well-established eqn (1) (Cm^3+^) and eqn (2) (Eu^3+^).^50–52^1
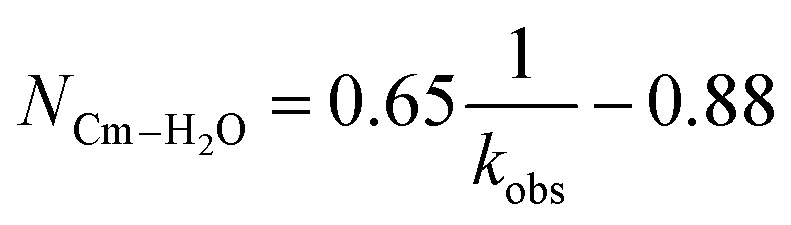
2
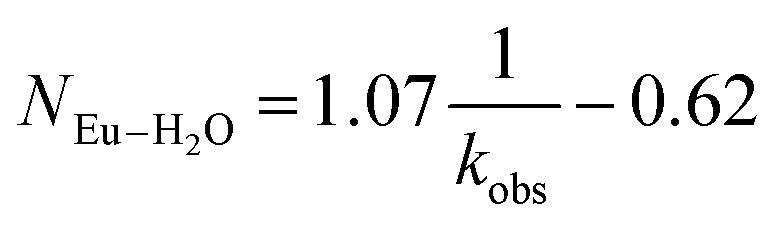


**Table 4:** Lifetimes (*k*_obs_), inverse lifetimes 
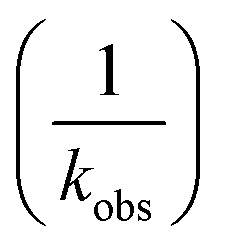
, and the hydration numbers (*N*_H_2_O_) extracted from exponential fitting of the observed kinetic decays obtained from solutions containing Cm^3+^ (0.3 mg, 1.20 µmol) dissolved in aqueous solutions of HOTf_(aq)_ (0.11 M), (NH_4_)O_2_CMe_(aq)_:HO_2_CMe_(aq)_ (0.1 M, 4 M, 15 M, pH 5)

Compound (matrix)	*k*_obs_ (ms)	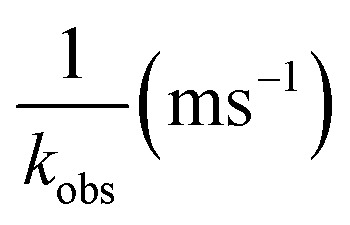	*N*_H_2_O_
Cm^3+^ HOTf_aq_ (0.11 M)	0.065(1)	15.4	9.1(5)
Cm^3+^ (NH_4_)O_2_CMe_(aq)_:HO_2_CMe_(aq)_ (0.1 M, pH 5.5)	0.068(1)	14.7	8.7(5)
Cm^3+^ (NH_4_)O_2_CMe_(aq)_:HO_2_CMe_(aq)_ (4 M, pH 5.5)	0.153(2)	6.54	3.4(5)
Cm^3+^ (NH_4_)O_2_CMe_(aq)_:HO_2_CMe_(aq)_ (15 M, pH 5.5)	0.244(2)	4.10	1.8(5)

**Table 5:** Lifetimes (*k*_obs_), inverse lifetimes 
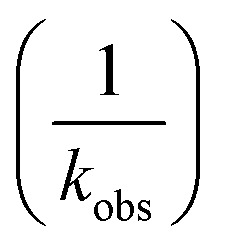
, and the hydration numbers (*N*_H_2_O_) extracted from exponential fitting of the observed kinetic decays obtained from solutions containing Eu^3+^ (0.5 mg, 3.3 µmol) dissolved in aqueous solutions of HOTf_(aq)_ (0.11 M), (NH_4_)O_2_CMe_(aq)_:HO_2_CMe_(aq)_ (0.1 M, 4 M, 15 M, pH 5)

Compound (matrix)	*k*_obs_ (ms)	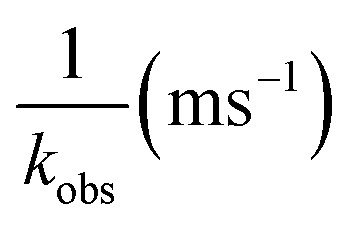	*N*_H_2_O_
Eu^3+^ HOTf_(aq)_ (0.11 M)	0.11(1)	9.09	9.0(5)
Eu^3+^ (NH_4_)O_2_CMe_(aq)_:HO_2_CMe_(aq)_ (0.1 M, pH 5.5)	0.19(3)	5.26	5.0(5)
Eu^3+^ (NH_4_)O_2_CMe_(aq)_:HO_2_CMe_(aq)_ (4 M, pH 5.5)	0.27(1)	3.70	3.3(5)
Eu^3+^ (NH_4_)O_2_CMe_(aq)_:HO_2_CMe_(aq)_ (15 M, pH 5.5)	0.45(2)	2.20	1.8(5)

By convention, the uncertainty associated with the TRFL determined hydration numbers was estimated at ±0.5.^33,50,51,53^ Consistent with previous studies, our analyses of the Cm^3+^- and Eu^3+^-aquo ions (HOTf, 0.11 M) showed hydration numbers at 9.1(5) and 9.0(5).^51^ At the other extreme, TRFL measurements made on single crystals of Cm(O_2_CMe)_5_^2−^ and Eu(O_2_CMe)_5_^2−^ were consistent with the X-ray single crystal data and showed small hydration numbers of 0.4(5) for Eu^3+^ and 0.8(5) for Cm^3+^. These results demonstrated that bound H_2_O provided the dominant deactivation pathway for excited Cm^3+^-aquo and Eu^3+^-aquo species, and established confidence that TRLF measurements would differentiate bound H_2_O from O_2_CMe^1−^.

The TRFL spectroscopic results from aqueous solutions provided evidence that increasing (NH_4_)O_2_CMe_(aq)_:HO_2_CMe_(aq)_ concentrations from 0.1 to 4 and 15 M substituted H_2_O for O_2_CMe^1−^ and decreased bound hydration numbers. For Cm^3+^, we measured 8.7(5) bound H_2_O ligands in dilute (NH_4_)O_2_CMe_(aq)_:HO_2_CMe_(aq)_ (0.1 M), which was equivalent to a hydration number of 8(1) determined by Cm^3+^ L_3_-edge EXAFS discussed above. In concentrated (NH_4_)O_2_CMe_(aq)_:HO_2_CMe_(aq)_, the TRFL measurements showed 1.8 ± 0.5 water molecules. This also agreed with the Cm^3+^ L_3_-edge EXAFS results, suggesting that the Cm^3+^ coordination sphere was dominated by coordinated O_2_CMe^1−^ in concentrated (NH_4_)O_2_CMe_(aq)_:HO_2_CMe_(aq)_ (15 M) with small contributions from bound H_2_O. The TRFL measurements at intermediate (NH_4_)O_2_CMe_(aq)_:HO_2_CMe_(aq)_ (4 M) concentrations indicated 3.5(0.5) water molecules bound by Cm^3+^. Assuming a total Cm^3+^ inner coordination number of 9 gave 2.8(5) bound O_2_CMe^1−^. Armed with this information, we decided that the best representation for the 4 M (NH_4_)O_2_CMe_(aq)_:HO_2_CMe_(aq)_ Cm^3+^ L_3_-edge EXAFS data involved the heteroleptic Cm(H_2_O)_5(1)_(O_2_CMe)_2(1)_^1(1)+^ species and we discarded the alternative Cm(H_2_O)_9(1)_^3+^ model discussed above.

## Outlook

The solution-phase speciation and solid-state structures from the +3 actinides (Ac, Am, Cm) have been described from the perspective of a combination of actinide L_3_-edge X-ray absorption spectroscopy, optical spectroscopy, and single crystal X-ray diffraction measurements. The results provided insight into what variables impact the complicated and dynamic conversion between the free An^3+^-aquo ions *vs.* An^3+^ cations complexed by acetate anions. Actinide-aquo ions dominated at one extreme; low concentration (NH_4_)O_2_CMe_(aq)_:HO_2_CMe_(aq)_ (0.1 to 0.2 M) buffered to an intermediate H^1+^ concentration (pH = 5.5). Prominence of the aquo ion maintained across the 5f-element series, from Ac^3+^ to Cm^3+^. Using actinide L_3_-edge EXAFS (for Cm^3+^) and time resolved fluorescence lifetime (TRFL) spectroscopy (for Cm^3+^ and Eu^3+^), we determined that increasing the (NH_4_)O_2_CMe_(aq)_:HO_2_CMe_(aq)_ (4 M) concentrations (also pH = 5.5) to intermediate levels increased the number of bound acetates, such that the dominant Cm^3+^ species present in solution were Cm(H_2_O)_5(1)_(O_2_CMe)_2(1)_^2+^ (assuming total coordination numbers of nine).

Pushing the (NH_4_)O_2_CMe_(aq)_:HO_2_CMe_(aq)_ concentration even higher (15 M) revealed two notable results. First, the total number of bound O_2_CMe^1−^ ligands increased. Second, the magnitude of the increase was dependent on the size of the actinide cation. For instance, actinide L_3_-edge XAFS and TRFL spectroscopy showed inner coordination spheres for Cm^3+^ and Eu^3+^ were dominated by approximately four bound O_2_CMe^1−^ ligands. In contrast, Ac^3+^ L_3_-edge EXAFS suggested that the dominant species for the larger Ac^3+^ cation (a weaker Lewis acid) contained only three acetates and was formulated as the neutral Ac(H_2_O)_6(1)_(O_2_CMe)_3(1)_. The observed reactivity differences can be rationalized by considering changes in Lewis acidity for the central actinide cations. The smaller, more Lewis acidic Cm^3+^ cation had a higher effective nuclear charge and attracted more O_2_CMe^1−^ anionic ligands than the larger Ac^3+^ cation.

These results directly refute the naïve assumption that chemistry for the +3 actinides is constant across the 5f-series and highlights a property unique to the f-elements that has been exploited for advancement of numerous technologies for decades (at least for the rare-earth elements, not actinides). For example, the rare earth elements are unique in that they represent a collection of sixteen metals (including Lu^3+^ and Y^3+^ and excluding Sc^3+^) whose properties are (in general) quite similar and whose +3 ionic radii methodically and subtly decrease as a function of element identity. This enables researchers to fine-tune properties for rare earth containing materials by judicious element selection, which is not possible for the main group and d-block transition element series. Examples range from modulating electronic properties in solid-state superconducting materials^54^ to tuning selectivity in diene polymerization.^55^ This slight difference in Lewis acidity has also provided a foundation for successful lanthanide separation technologies, like those that were developed by Hoffman, Choppin, and Spedding and relied on systematically changing the concentration of a complexing agent (like hydroxy isobutyric acid) during ion exchange chromatography.^56–59^ These same properties have provided a basis for separation of adjacent minor actinides as well (Am^3+^ from Cm^3+^).^60^

Our acetate studies align with the assumption (and a limited number of experimental observations) that actinide(iii) complexation is directly influenced by actinide Lewis acidity. It provides a rare example showing how 5f-element speciation varies as actinide ionic radii contract, which is analogous to that observed for rare earth elements. Although this concept is expected, the impact from an “actinide contraction” on 5f-element coordination chemistry is scarce in the literature. The absence is especially obvious for actinium and the transuranic actinides (like Am and Cm), owing to the challenges associated with obtaining and studying these rare and radioactive elements. The implications of An^3+^ speciation differences in (NH_4_)O_2_CMe_(aq)_:HO_2_CMe_(aq)_ buffered solutions are subtle, but important. They highlight that the dominant species present in high concentration (NH_4_)O_2_CMe_(aq)_:HO_2_CMe_(aq)_ buffered solutions for the early actinides is not equivalent to that for the minor actinides. We speculated that the increased complexation tendencies for the late actinide(iii) cations *vs.* the early actinides likely persists even when the (NH_4_)O_2_CMe_(aq)_:HO_2_CMe_(aq)_ concentration is less than 15 M. For instance, it seems possible that acetate likely prefers binding small f-elements (like Cm^3+^) over large actinides (like Ac^3+^) between 4 and 15 M (NH_4_)O_2_CMe_(aq)_:HO_2_CMe_(aq)_ concentrations.

Fully characterizing how small changes in Lewis acidity impact aqueous complexation chemistry in (NH_4_)O_2_CMe_(aq)_:HO_2_CMe_(aq)_ buffered solutions, and in other relevant aqueous matrixes, could have widespread impact. There is potential to substantially expand fundamental chemical understanding for actinide cations, especially those that are highly radioactive and difficult to study (like Ac^3+^ and Cm^3+^). Better characterizing this aspect of aqueous actinide coordination chemistry would arm researchers with critical information that touches on virtually every aspect of relevant actinide science and technology; spanning from advanced environmental fate and transport models to designing new technologies that selectively deliver actinide cations to diseased tissue for targeted alpha therapeutic applications. It is our considered opinion that the study herein complements influential campaigns reported previously, those focused on better defining the fundamental landscape of actinide speciation in the presence of simple organic and inorganic complexing agents in aqueous media.^10,17,36,40,61–64^ We hope that collectively this body of work allures new research groups into the area and motivates additional study of actinides in relevant aqueous solutions.

## Methods

### General consideration

**Caution!** The ^245^Cm [half-life = *t*_1/2_ = 8423(74) years],^65^^246^Cm [*t*_1/2_ = 4706(40) years],^65^^247^Cm [*t*_1/2_ = 1.56(5) × 10^7^ years],^65^^248^Cm [*t*_1/2_ = 3.48(6) × 10^5^ years],^65^^243^Am [*t*_1/2_ = 7364(22) y],^65^ and ^227^Ac [*t*_1/2_ = 21.772(3) years]^65^ isotopes – and their daughters – present serious health threats due to their neutron-, α-, β-, and γ-emissions. Hence, all studies that involved manipulation of these isotopes were conducted in a radiation laboratory equipped with HEPA filtered hoods, continuous air monitors, negative pressure gloveboxes, and monitoring equipment appropriate for neutron-, α-, β-, and γ-particle detection. Entrance to the laboratory space was controlled with a hand and foot monitoring instrument for α-, β-, and γ-emitting isotopes and a full body personal contamination monitoring station. Free-flowing solids were handled within negative pressure gloveboxes equipped with HEPA filters. The ^248^Cm, ^243^Am, and ^227^Ac isotopes were supplied by the United States Department of Energy Office of Science Isotope Program in the Office of Nuclear Physics. Oxidation state and chemically pure Ac^3+^ stock solutions were prepared as previously described.^36,66^ Note, the ^248^Cm sample used in this study contained small amounts of radioisotopic contamination; ^248^Cm (86.519% by weight), ^247^Cm (0.133% by weight), ^246^Cm (12.8605 by weight), and ^245^Cm (0.488% by weight). The Am and Cm stock solutions were purified by liquid extraction chromatography with a DGA resin charged Bio-Rad column (*vide infra*). Optima grade acetic acid was obtained commercially (Fisher Scientific). The Eu used in spectroscopy and synthesis efforts was purchased from Sigma Aldrich as the EuCl_3_·6H_2_O (trace metal basis). Water used for ^243^Am and ^248^Cm in these experiments was deionized and passed through a Barnstead water purification system until a resistivity of 18 MΩ was achieved. For ^227^Ac, the water was purified further by distillation using a Teflon distilling apparatus, which reduces trace metal contamination. The ammonium acetate/acetic acid [(NH_4_)O_2_CMe:HO_2_CMe, 0.1 M] buffered solution was prepared by dissolving ammonium acetate (5.78 g, 0.075 mole) in water (5 mL, 18.2 MΩ cm^−1^ resistivity and Teflon distilled) within a polyethylene falcon tube (15 mL). Using pH paper, the pH for this solution was then adjusted to 5.5 using glacial acetic acid (17.4 M). The ammonium hydroxide solution [14.5 M, NH_4_OH_(aq)_, Optima Grade], was acquired from Fisher Chemical.

Single crystal UV-vis-NIR measurements were made on single crystals mounted on a quartz slide under oil using a Craic Technologies microspectrophotometer. For absorbance measurements, data were collected from 9090.91 to 40 000 cm^−1^ (1100 nm to 250 nm).

Solution-phase UV-vis-NIR measurements were recorded on a Varian Cary 6000i spectrophotometer in a screw cap quartz cuvette.

### Bisammonium europium(iii) pentakisacetate, (NH_4_)_2_Eu(O_2_CMe)_5_

In an open front hood and with no attempt to exclude air and moisture, europium(iii) trisnitrato hexahydrate [Eu(NO_3_)_3_·6H_2_O, 15 mg, 0.04 mmol] was dissolved in H_2_O (18 mΩ, 2 mL). Europium(iii) was then precipitated by adding ammonium hydroxide, NH_4_OH_(aq)_ (14.5 M, 1 mL). The supernatant was removed by centrifugation (3000 rpm, 3 min), the pellet suspended in H_2_O (18 MΩ, ∼5 mL) using a stir rod, and the resulting supernatant removed again by centrifugation. Using this procedure, the pellet was washed a total of three times. Then the washed pellet was dissolved in (NH_4_)O_2_CMe_(aq)_ (10 M, 3 mL). Aliquots (0.5 mL) from this stock solution were transferred to six crystallization vials and colorless single crystals (rectangular platelets) suitable for single crystal X-ray diffraction were isolated after 2.5 weeks of slow-evaporation (0.65 mg, 25.9% yield).

### Preparation of actinide stock solutions and recovery (general)

Both solution and solid-state 5f-element syntheses relied on preparing chemically pure stock solutions of Ac^3+^_(aq)_, Am^3+^_(aq)_, and Cm^3+^_(aq)_. Initially, these solutions were generated according to our previous reports,^36^ with the exception that prior to purification samples were fired at 850 °C to remove the organic contaminants. However, for Am^3+^ and Cm^3+^ the procedures evolved over the course of this study. We switched from using DOWEX (50WX8 50–100 mesh) cation exchange resin to the extraction resin (branched DGA 100–200 mesh from Eichrom) deployed in Ac^3+^ recovery and purification.^36^ The change was primarily driven by higher Am^3+^ and Cm^3+^ recoveries from DGA (>98%) *vs.* cation exchange resins (∼80%). The modest increase in yield was of particular significance for these isotopes, given their rarity and value. After using the Ac^3+^, Am^3+^, and Cm^3+^ stock solutions in the coordination chemistry experiments described below, these +3 actinides were recovered using a variation of the procedure described above for preparing stock solutions. Firing at 850 °C was skipped because Am^3+^ and Cm^3+^ could be separated from the majority of the acetate and acetic acid by precipitation with HF. More rigorous purification was achieved with the DGA column. For Ac^3+^, which was present on the microscopic level (µg), the HF precipitation was not possible. Hence, Ac^3+^ purifications combined the DGA extraction chromatography with anion exchange chromatography using AG-1X8 resin.^67^

### Preparation of curium(iii) stock solution

**Scheme 3:**
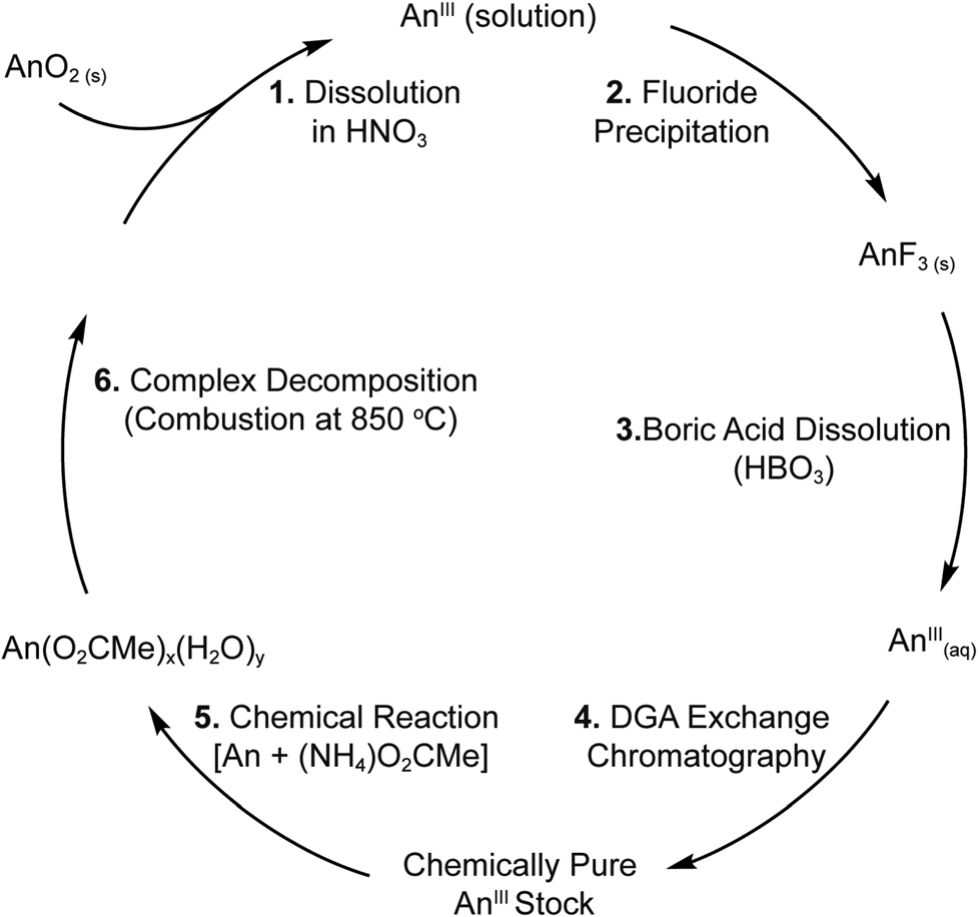
Flow diagram of the processing and purification of An^3+^ (An = Cm, Am) in aqueous media.

In a HEPA filtered open front fume hood and with no attempt to exclude air and moisture, a Cm^3+^ stock solution was prepared as shown in Scheme 3. Residues known to contain ^248^Cm (3 to 10 mg) that had been used in previous experimental campaigns were fired in a muffle furnace (a ramp rate of 1 °C min^−1^ was used to first heat to 110 °C to dehydrate the sample for 120 minutes, before ramping at 0.5 °C min^−1^ to 850 °C where it was held for 180 minutes) within a quartz beaker. The residue was dissolved in nitric acid (HNO_3_, 8 M, 2.5 mL) and transferred into a falcon tube (50 mL). Hydrofluoric acid (HF, 28 M, 5 mL) was added and a faint yellow solid precipitated. After 15 min, the suspension was centrifuged and the supernatant (colorless) was removed leaving behind a pale-yellow pellet. The pellet was washed with water (18 MΩ, 2 × 5 mL). Then, the pellet was dissolved in the following way. Note, curium trifluoride (CmF_3_) can be difficult to dissolve. Using a stir rod to agitate the pellet, boric acid [H_3_BO_3(aq)_, saturated, 2 mL] was added and the sample was heated at approximately 80 °C (5 min). Next, HNO_3(aq)_ (8 M, 1 mL) was added. After heating (80 °C; 5 min), H_3_BO_3(aq)_ (saturated, 1 mL) was added and the mixture heated for another 5 min. A final aliquot of HNO_3(aq)_ (8 M, 1 mL) was added and the samples heated near 80 °C for another 5 min. At this point, the solid had completely dissolved and the HNO_3_ concentration for the solution was 4 M.

A Bio-Rad column (10 mL) was charged with DGA resin (Eichorm, 50–100 µm, 3 mL). The resin was conditioned with HNO_3(aq)_ (4 M, 3 × 5 mL), H_2_O (18 MΩ, 5 mL), and HCl (0.1 M, 3 × 5 mL). The column was then washed once with HNO_3(aq)_ (4 M, 5 mL) and the Cm^3+^ solution in HNO_3(aq)_ (4 M, 5 mL) was loaded onto the column. Under these conditions, Cm^3+^ was retained on the resin. The column was washed with HNO_3(aq)_ (4 M, 3 × 5 mL). Then, Cm^3+^ was eluted with HCl (0.1 M, 8 × 1 mL). The Cm^3+^ elution profile was quantified by stippling small volumes (one drop) of the eluted fraction onto Pyrex slides. After the samples dried under air, gross ^248^Cm α-activity was quantified by analyzing each slide using a Ludlum 3939E α-, β-, γ-stationary survey instrument (for low activity samples) or a handheld portable α-survey meter (Ludlum 139) for high activity samples. Afterwards, stippled ^248^Cm was recovered by soaking the slides in HCl (6 M) and set aside for reprocessing at a later date. The second, third, and fourth Cm^3+^ elution fractions (which contained the majority of the activity) were combined and the solution was heated to a soft dryness. The resulting residue was dissolved in HCl_(aq)_ (0.1 M, 1.0 mL), giving a chemically and radiochemically pure Cm^3+^ stock solution. The Cm^3+^ concentration was determined by analyzing an aliquot (250 µL) of the stock solution in trifluoromethanesulfonic acid (HOTf_(aq)_, 0.11 M, 0.75 mL) by UV-vis spectroscopy. For these measurements, we assumed the 25 220.7 cm^−1^ (396.5 nm) absorbance had an extinction coefficient of 52.9 L mol^−1^ cm^−1^, as previously reported.^68,69^

### Bisammonium curium(iii) pentakisacetate, (NH_4_)_2_Cm(O_2_CMe)_5_

In an open front hood and with no attempt to exclude air and moisture, an aliquot of the Cm stock solution (34.4 mM, 0.1 mL), described above, was transferred into a Falcon tube (50 mL). Curium(iii) was precipitated by adding NH_4_OH(aq) (14.5 M, 1 mL) and the curium title compound was prepared as described above for (NH_4_)_2_Eu(O_2_CMe)_5_. Pale yellow crystals (rectangular plates) suitable for single crystal X-ray diffraction were obtained after 2.5 weeks by slow evaporation from an (NH_4_)O_2_CMe_(aq)_ (10 M, 0.5 mL) solution.

### Preparation of americium(iii) stock solution

In an open front hood and with no attempt to exclude air and moisture, americium(iv) oxide (AmO_2_, 50 mg of Am, 0.206 mmol), acquired from Oak Ridge National Laboratory, was dissolved in HNO_3_ (4 M, 2.5 mL). The solution was evaporated to a soft dryness and the resulting residue dissolved with HNO_3_ (4 M, 2.5 mL). This was repeated three times to ensure complete removal of Cl^1−^. The resulting americium nitrate solution was used to prepare an Am^3+^ stock solution in direct analogy to the procedure described above for the Cm. This was achieved by precipitating AmF_3_ (using HF) followed by extraction chromatography with the DGA resin. Note, five columns were prepared and the Am solution was split so that 10 mg of Am were run through each column. Quantification of Am^3+^ eluting from the DGA column was carried out using γ-spectroscopy, as opposed to monitoring activity of aliquots stippled on Pyrex slides as described for Cm above. The final Am^3+^ stock solution was isolated in 95.7% yield and contained 47.9 mg of Am^3+^ dissolved in 3 mL of HCl (0.1 M), as determined by a combination of γ-spectroscopy and UV-vis. For these γ- and UV-vis measurements, we assumed the γ-peak at 74.7 keV had a branching ratio of 67.2% (ref. 65) and the absorbance at 19 872.8 cm^−1^ (503.2 nm) had an extinction coefficient of 410 mol^−1^ cm^−1^,^70^ respectively.

### Bisammonium americium(iii) pentakisacetate, (NH_4_)_2_Am(O_2_CMe)_5_

In an open front hood and with no attempt to exclude air and moisture, an aliquot of the Am^3+^ stock solution (0.165 mM, 2.5 mL), described above, was transferred into a Falcon tube (50 mL). Americium(iii) was precipitated by adding NH_4_OH_(aq)_ (14.5 M, 1 mL) and the americium title compound was prepared as described above for (NH_4_)_2_Eu(O_2_CMe)_5_. Pale peach crystals (rectangular plates) suitable for single crystal X-ray diffraction were obtained after 1 week by slow evaporation from an (NH_4_)O_2_CMe_(aq)_ (10 M, 0.25 mL) solution.

### Single crystal X-ray diffraction

Single crystals of (NH_4_)_2_M(O_2_CMe)_5_ (M = Am, Cm) were mounted with three appropriate layers of containment prior to single-crystal X-ray diffraction studies, as previously described.^66^ All other single crystals were mounted on nylon loops with mineral oil (Hampton Research). Diffraction data were obtained using a D8 Bruker QUEST diffractometer. No corrections for crystal decay were necessary. Standard Apex III software was used for determination of the unit cells and data collection control. The intensities of the reflections of a sphere were collected by combining 13 sets of exposures (frames), which totaled to 2035 frames with an exposure time of 15 per frame, depending on the crystal. Apex III software was used for data integration including Lorentz and polarization corrections. All crystal structures were solved using SHELX software,^71^ and PLATON^72^ was used to check the Crystallographic Information Files (CIFs) for missed symmetry and twinning. The CIFs used in this manuscript are available through the Cambridge Crystal Data Centre (CCDC; 2016262, 2016263, 2016264, 2016265).

### Solution-phase sample preparation

Solutions for UV-vis (Cm), fluorescence (Cm), and XAS (Cm and Ac) spectroscopy measurements were prepared in an open front fume hood with no attempt to exclude air and moisture. Three buffered solutions, 0.1 M, 4 M, and 15 M, of acetate concentrations, HO_2_CMe:(NH_4_)O_2_CMe (pH = 5.5) were prepared. These samples were prepared by adding aliquots of the purified Cm^3+^ (0.7 mg, 2.81 µmol for XAS; 0.3 mg, 1.20 µmol for UV-vis) and Ac^3+^ (28 µg, 0.123 µmol) stock solutions into conical glass vials. The aqueous solution was removed by heating the samples on a hot plate around 110 °C under a flow of air until a soft dryness was achieved. These residues were dissolved in (NH_4_)O_2_CMe:HO_2_CMe buffered solutions described above and the solutions were transferred to XAS holders or cuvettes (for UV-vis) for spectroscopic analyses.

### Emission and time resolved fluorescence lifetime measurements

The emission spectra and lifetimes were obtained using a Photon Technologies International (PTI) model QM-04 fluorometer with Felix32 software. Steady state excitation and emission spectra were collected using a 75 W Xe arc lamp as an excitation source and a thermoelectrically cooled Hamamatsu R928 photomultiplier tube to measure emission at a 90° angle. Excitation and emission slits were set to give a 1.5 nm bandpass for all samples, with the exception of the solid (NH_4_)_2_Eu(O_2_CMe)_5_ and Eu(H_2_O)_9_(OTf)_3_. In these cases, the bandpass was set to 3 nm. Lifetimes were collected using a µs Xe flash lamp (Xenoflash) operated at 5 Hz for the excitation source and a time-gated PMT detection system oriented at a 90° angle. Again, the bandpass was set to 1.5 nm for all samples except solid (NH_4_)_2_Eu(O_2_CMe)_5_ and Eu(H_2_O)_9_(OTf)_3_, where a 3 nm bandpass was used. The raw data from the PTI Quanta were exported into Origin 2019. Then, the data were fit with a double exponential decay function (IRF and decay), from which kinetic lifetimes were extracted. Radiological samples were contained using screw-top quartz cuvettes. Cuvettes were loaded in a HEPA filtered open-front hood and surveyed using a Ludlum 3030 smear counter before transport to the instrument.

### Radiological containment for XAS samples

The custom-made XAS holders and handling procedures provided adequate containment (three layers) and administrative/engineering controls that guarded against release of radiological material during shipment and data acquisition. The holder consisted of a Teflon body with a 5 mm well for Cm^3+^ and a 2 mm well for Ac^3+^ equipped with a set of Teflon windows (1 mil) and a Kapton window (1 mil). Solutions were introduced into the holder through an injection hole sealed with a Teflon gasket that was held in place by an aluminum plate. This primary holder was held within a secondary container, which in turn was nested within the tertiary container. The secondary and tertiary containers were best described as a set of aluminum holders equipped with Kapton windows (2 mil) and rubber gaskets.

### XAS data acquisition

The X-ray absorption spectra (XAS) were collected at the Stanford Synchrotron Radiation Lightsource (SSRL) under dedicated operating conditions (3.0 GeV, 5%, 500 mA) on end station 11-2. This beamline was equipped with a 26-pole and a 2.0 Tesla wiggler. Using a liquid nitrogen-cooled double-crystal Si(220) (*Φ* = 90° and 0° for Ac and Cm, respectively) monochromator and employing collimating mirrors, a single energy was selected from the incident white beam. Vertical acceptance was controlled by slits positioned before the monochromator. All measurements were conducted with the monochromator crystals fully-tuned. For these experiments, higher harmonics from the monochromatic light were removed using a 370 mm Rh coated harmonic rejection mirror. The Rh coating was 50 nm with 20 nm seed coating and the substrate was Zerodur. The harmonic rejection cut-off was set at 21 700 eV by the mirror angle, thereby controlling which photons experience total external reflection.

The samples were attached to the beamline 11-2 XAS rail. The rail was equipped with three ionization chambers through which nitrogen gas continually flowed. One chamber (10 cm long) was positioned before the sample holder, to monitor the incident radiation (*I*_0_). The second chamber (30 cm long) was positioned after the sample holder, such that sample transmission (*I*_1_) could be evaluated against *I*_0_, while a third chamber (*I*_2_, 30 cm long) positioned downstream from *I*_1_ so that the XANES of a calibration foil could be measured *in situ* during the XAS experiments against *I*_1_. All actinide L_3_-edge XAS samples were measured by monitoring sample fluorescence against the incident radiation (*I*_0_). An additional 100 element Ge fluorescence detector was positioned at 90° to the incident radiation (*I*_0_) and windowed on either the Cm Lα_1_-emission line (14 961 eV) or the Ac Lα_1_-emission line (12 652 eV). With this designation, actinide L_3_-edge XAS spectra (15 871 eV and 18 970 eV for Ac and Cm, respectively) were recorded in fluorescence mode as the ratio of fluorescence intensity over the intensity of the incident radiation (*I*_0_). High-energy contributions to the fluorescence signal were removed by equipping the Ge detector with Soller slits and either a Sr filter (3 absorption lengths for Cm) or a Br filter (3 absorption lengths for Ac).

### XAS data analysis

Data manipulations and analyses were conducted as previously described.^36,40^ The Ac^3+^ L_3_-edges XAS data were calibrated to the Rb K-edge (15 200 eV) from a RbCl pellet diluted in BN to 1 absorption length and Cm^3+^ L_3_-edges XAS spectra were calibrated to the Zr K-edge from a Zr metal-foil (18 013.3 eV). All calibration samples were measured *in situ*. To correct for detector dead time, nonlinear response curves were defined from 0 to ∼70% dead (windowed counts of the emission line *versus* the total incoming counts into the solid-state detector) using an Y 3 mm filter (∼400 above the Y K-edge) for Cm and a Se filter (∼400 eV above the Se K-edge) for Ac. Each channel was manually surveyed for outliers, which were omitted. The deadtime correction was applied before averaging the individual channels. Then, the 8 individual scans were aligned with the *in situ* calibration foil and averaged using IFEFFIT^73^ within the Athena software package. The XAS data were analyzed by fitting a line to the pre-edge region, which removed the background from experimental data in the spectra. Then, a third-order polynomial fit was chosen for the post-edge region. The difference between pre- and post-edge lines was set to unity at the first inflection point, normalizing the absorption jump to 1.0. To remove contributions from low frequency noise, a spline function was fit over the absorption background of an isolated atom and subtracted from the data. The EXAFS data were then analyzed by shell-by-shell fitting methods using IFEFFIT^73^ software and FEFF8 calculations.^74,75^ The spectra were *k*^3^-weighted and Fourier transformed prior to non-linear least squares curve fitting. The energy phase shift parameter (Δ*E*_o_) was refined as a global parameter and then fixed for the remainder of the curve-fitting analyses. The amplitude reduction factor (*S*_0_^2^) was set to 0.9 in accordance with our previous An–Cl, An-aquo, An–NO_3_ studies and numerous other actinide EXAFS reports.^34,35^ The actinide coordination number (*N*), scattering path length (*R*), and mean-squared displacements (*σ*^2^), were used as variables. Values from *N*, *R* and *σ*^2^ were refined as free variables. In the Cm^3+^ case, atomic coordinates for the FEFF8 calculations were obtained from the single-crystal X-ray data (CIFs) for Cm(O_2_CMe)_5_^2−^ (described above). For Ac^3+^, the central Cm^3+^ cation was substituted *in silico* to generate a hypothetical Ac(O_2_CMe)_5_^2−^.

## Author contributions

Zachary R. Jones, Maryline G. Ferrier, Benjamin W. Stein, Laura M. Lilley, and Stosh A. Kozimor conceived the study. Zachary R. Jones Maksim Y. Livshits, Benjamin W. Stein, and Stosh A. Kozimor wrote the original draft of the paper. All authors contributed to revising that document. Maryline G. Ferrier and Laura M. Lilley contributed to XAS sample preparation alongside Benjamin W. Stein and Stosh A. Kozimor. Zachary R. Jones, Maryline G. Ferrier, Jennifer N. Wacker, David H. Woen and Laura M. Lilley made the XAS measurements and Zachary R. Jones and Stosh A. Kozimor analyzed the data. Elodie Dalodière and Veronika Mocko contributed to actinide purification and recovery. Frankie D. White, Karah E. Knope, Brian L. Scott, and Jennifer N. Wacker contributed by making the single crystal X-ray absorption measurements. Maksim Y. Livshits and Benjamin W. Stein led efforts associated with the optical measurements.

## Conflicts of interest

The authors have no conflicts of interest with this work.

## Supplementary Material

SC-012-D1SC00233C-s001

SC-012-D1SC00233C-s002
