# Supplementary material for: Advancing understanding of actinide(iii) (Ac, Am, Cm) aqueous complexation chemistry
Source: Chem Sci. 2021 Mar 17;12(15):5638–54. doi: 10.1039/d1sc00233c (PMC8179631; doi:10.1039/d1sc00233c)
Supplement: SC-012-D1SC00233C-s001 [file SC-012-D1SC00233C-s001.pdf]

Electronic Supplementary Material (ESI) for Chemical Science.
This journal is © The Royal Society of Chemistry 2021

Supporting information

Advancing Understanding of Actinide(III) (Ac, Am, Cm) Aqueous Complexation Chemistry

Zachary R. Jones,[1] Maksim Y. Livshits,[1] Frankie D. White,[1] Elodie Dalodière,[1] Maryline G. Ferrier,[1] Laura M. Lilley,[1] Karah E. Knope,[2] Stosh A. Kozimor,[1,*] Veronika Mocko,[1] Brian L. Scott,[1] Benjamin W. Stein,[1,*] Jennifer N. Wacker,[1,2] David H. Woen.[1]

[1] Los Alamos National Laboratory (LANL), P.O. Box 1663, Los Alamos, New Mexico, 87545, USA

[2] Department of Chemistry, Georgetown University, 37th and O Streets NW, Washington, D.C. 20057, USA

* To whom correspondence should be directed*, e-mail; stosh@lanl.gov, bstein@lanl.gov*

	**Table of Contents**	**Page**
**Fig. S1**	Previously published Ac-aquo L_3-edge XANES	3
**Fig. S2**	Previously published Ac-aquo ion L_3-edge XANES with I_o	4
**Fig. S3**	Solid-state structure of **$(NH_4)_2Eu(O_2CMe)_5$**	5
**Table S1**	Crystal structure data for **$(NH_4)_2Eu(O_2CMe)_5$**	6
**Fig. S4**	Solid-state structure of **$(NH_4)_2Am(O_2CMe)_5$**	7
**Table S2**	Crystal structure data for **$(NH_4)_2Am(O_2CMe)_5$**	8
**Fig. S5**	Solid-state structure of **$(NH_4)_2Cm(O_2CMe)_5$**	9
**Table S3**	Crystal structure data for **$(NH_4)_2Cm(O_2CMe)_5$**	10
**Table S4**	Tabulated Data for Fig.s **S1** and **S2**	11

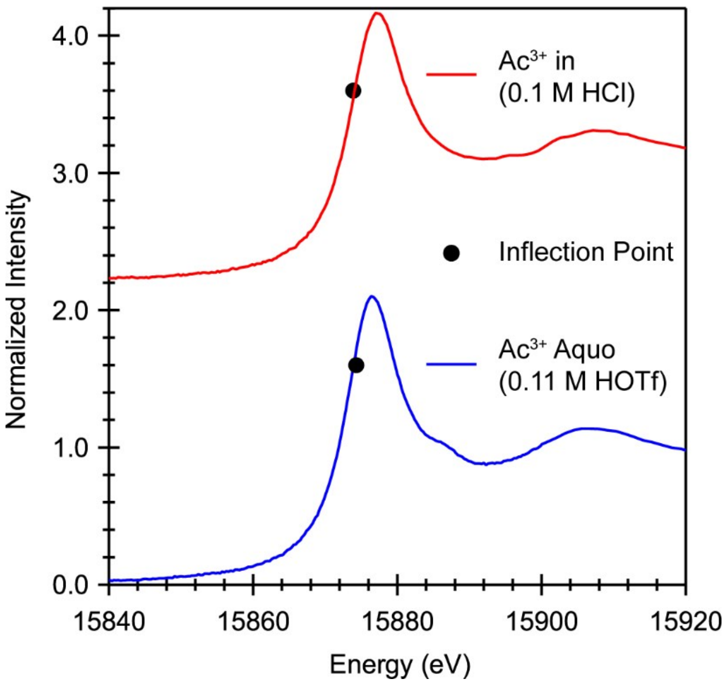

Fig. S1 Actinium L_3-edge X-ray absorption spectra from (blue) Ac^{3+}-aquo in 0.11 M HOTf (data published in reference Ferrier, *et al. Nature Comm.* ***2016****, 7, 12312*), and (red) Ac^{3+} in 0.1 M HCl (data published in reference Ferrier, *et al. ACS Central Sci.* ***2017****, 3, 176*). Note, there is an I0 glitch near 15886 in the Ac^{3+}-aquo in 0.11 M HOTf data.

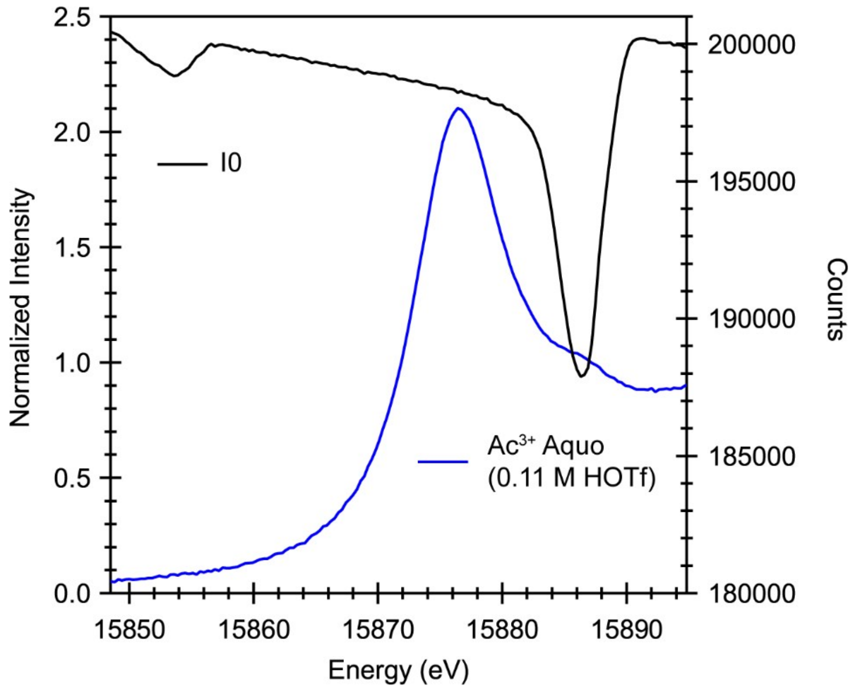

Fig. S2 Actinium L_3-edge X-ray absorption spectra from (blue) Ac^{3+}-aquo in 0.11 M HOTf (data published in reference Ferrier, *et al. Nature Comm.* ***2016****, 7, 12312*), and the counts corresponding to the incident beam (I_o) highlighting the dip in intensity resulting from the monochromator crystal glitch around 15886 eV.

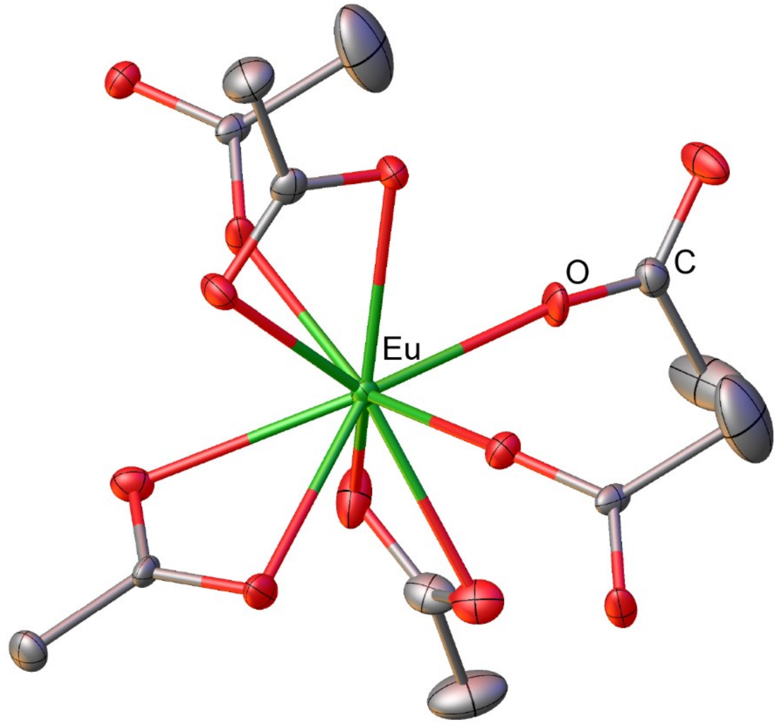

Fig. S3 A thermal ellipsoid plot generated form the single crystal X-ray diffraction data of $(NH_4)_2Eu(O_2CMe)_5$ (green = Eu, red = O, gray = C). The thermal ellipsoids have been plotted at the 50% probability. Hydrogen atoms and the NH_4^{1+} cations have been omitted.

Table S1 Crystal data for $(NH_4)_2Eu(O_2CMe)_5$.

Identification code	CCDC#2016263
Empirical formula	$EuO_{11}N_2C_{10}H_{17}$
Formula weight	493.22
Temperature/K	296.15
Crystal system	monoclinic
Space group	$P2_1/n$
a/Å	6.7137(4)
b/Å	14.8928(8)
c/Å	20.2772(12)
α/°	90
β/°	97.157(3)
γ/°	90
Volume/Å^3	2011.6(2)
Z	4
ρ_{calc}g/cm^3	1.629
μ/mm^{-1}	3.166
F(000)	968.0
Crystal size/mm^3	0.75 × 0.30 × 0.30
Radiation	MoKα (λ = 0.71073)
2Θ range for data collection/°	3.402 to 56.426
Index ranges	$-8 \leq h \leq 8$, $-19 \leq k \leq 19$, $-26 \leq l \leq 26$
Reflections collected	63722
Independent reflections	4950 [R_{int} = 0.0898, R_{sigma} = 0.0533]
Data/restraints/parameters	4950/0/225
Goodness-of-fit on F^2	1.181
Final R indexes [I>=2σ (I)]	R_1 = 0.0584, wR_2 = 0.1198
Final R indexes [all data]	R_1 = 0.0716, wR_2 = 0.1241
Largest diff. peak/hole / e Å^{-3}	2.16/-2.54

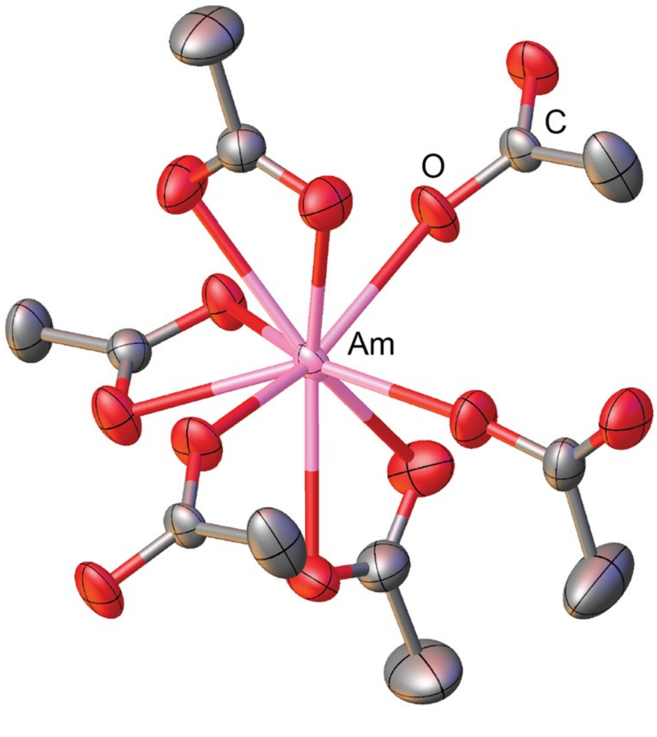

Fig. S4 A thermal ellipsoid plot generated form the single crystal X-ray diffraction data of $(NH_4)_2Am(O_2CMe)_5$ (pink = Am, red = O, gray = C). The thermal ellipsoids have been plotted at the 50% probability. Hydrogen atoms and the NH_4^{1+} cations have been omitted.

Table S2 Crystal data for $(NH_4)_2Am(O_2CMe)_5$.

Identification code	CCDC#2016262
Empirical formula	$AmO_{11}N_2C_{10}H_{17}$
Formula weight	582.52
Temperature/K	298.39
Crystal system	monoclinic
Space group	$P2_1/n$
a/Å	6.7963(3)
b/Å	15.0543(8)
c/Å	20.4812(11)
α/°	90
β/°	97.2970(10)
γ/°	90
Volume/Å^3	2078.53(18)
Z	4
ρ_{calc}g/cm^3	1.884
μ/mm^{-1}	3.739
F(000)	1219.0
Crystal size/mm^3	0.8 × 0.3 × 0.3
Radiation	MoKα (λ = 0.71073)
2Θ range for data collection/°	5.412 to 67.466
Index ranges	-10 ≤ h ≤ 10, -23 ≤ k ≤ 23, -31 ≤ l ≤ 31
Reflections collected	47585
Independent reflections	8271 [R_{int} = 0.0382, R_{sigma} = 0.0250]
Data/restraints/parameters	8271/0/225
Goodness-of-fit on F^2	1.090
Final R indexes [I>=2σ (I)]	R_1 = 0.0284, wR_2 = 0.0719
Final R indexes [all data]	R_1 = 0.0367, wR_2 = 0.0749
Largest diff. peak/hole / e Å^{-3}	2.37/-0.87

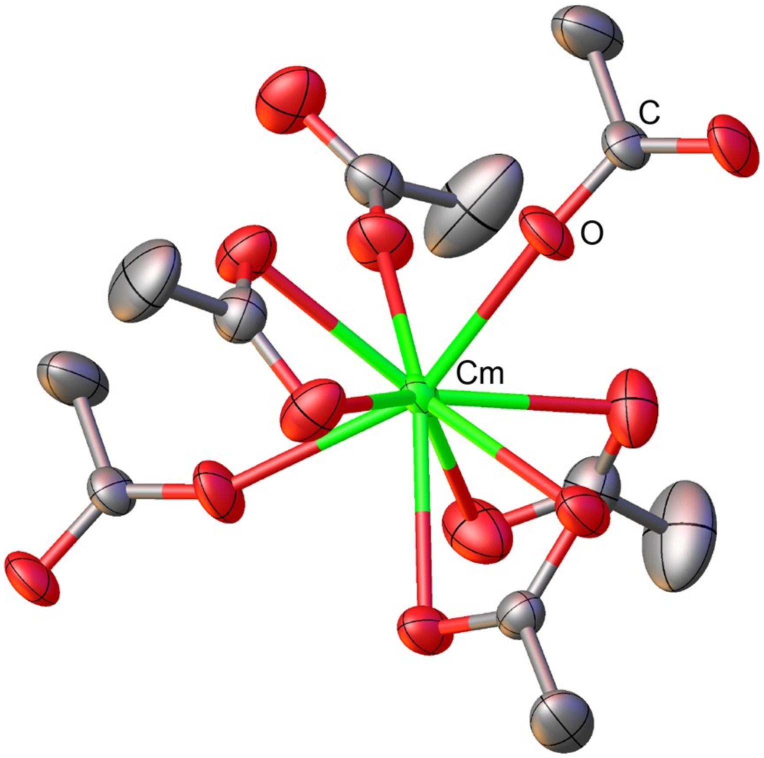

Fig. S5 A thermal ellipsoid plot generated form the single crystal X-ray diffraction data of $(NH_4)_2Cm(O_2CMe)_5$ (green = Cm, red = O, gray = C). The thermal ellipsoids have been plotted at the 50% probability. Hydrogen atoms and the NH_4^{1+} cations have been omitted.

Table S3 Crystal data for $(NH_4)_2Cm(O_2CMe)_5$.

Identification code	CCDC#2016265
Empirical formula	$CmO_{11}N_2C_{10}H_{25}$
Formula weight	593.51
Temperature/K	298.06
Crystal system	monoclinic
Space group	$P2_1/n$
a/Å	6.7801(3)
b/Å	15.0056(7)
c/Å	20.4890(10)
α/°	90
β/°	97.2400(10)
γ/°	90
Volume/Å^3	2067.92(17)
Z	4
ρ_{calc}g/cm^3	1.880
μ/mm^{-1}	3.989
F(000)	1100.0
Crystal size/mm^3	0.2 × 0.15 × 0.1
Radiation	MoKα (λ = 0.71073)
2Θ range for data collection/°	5.43 to 56.542
Index ranges	$-9 \leq h \leq 9$, $-19 \leq k \leq 20$, $-27 \leq l \leq 27$
Reflections collected	32150
Independent reflections	5093 [R_{int} = 0.0321, R_{sigma} = 0.0193]
Data/restraints/parameters	5093/0/225
Goodness-of-fit on F^2	1.171
Final R indexes [I>=2σ (I)]	R_1 = 0.0252, wR_2 = 0.0638
Final R indexes [all data]	R_1 = 0.0289, wR_2 = 0.0652
Largest diff. peak/hole / e Å^{-3}	1.82/-0.73

Table S4 Tabulated Data from Fig. S1-S2.

Energy (eV)	Ac in 0.11 M HOTf	I0 Ac in 0.11 M HOTf	Energy (eV) Ac in 0.1 M HCl	Ac in 0.1 M HCl
15639.8	0.0126594	205884	15468.1	0.0280115
15645.7	0.00481276	205890	15473.9	0.0257753
15651.6	0.00293112	205746	15479.8	0.0243976
15657.4	0.00391762	205510	15485.7	0.0287314
15663.3	-0.00470631	205356	15491.6	0.0273089
15669.2	0.00964801	205144	15497.5	0.0242683
15675.1	0.000913579	205428	15503.4	0.0167116
15681	0.00604719	206384	15509.2	0.0287685
15686.9	-0.000612236	206350	15515.1	0.0198663
15692.8	-0.00643875	206404	15521	0.0198588
15698.6	-0.00285543	206066	15526.9	0.0158353
15704.5	-0.0027566	205908	15532.8	0.0153119
15710.4	-0.000259465	205798	15538.6	0.0145092
15716.3	-0.00184415	205582	15544.5	0.0131751
15722.2	-0.000569398	205354	15550.4	0.0074762
15728	-0.00572285	205124	15556.3	0.0137902
15733.9	-0.00503089	204926	15562.2	0.0151856
15739.8	-0.00313219	204676	15568.1	0.00872356
15745.7	0.000627347	204494	15573.9	0.013068
15751.6	-0.00114518	204278	15579.8	0.00603819
15757.5	-0.000936867	203726	15585.7	0.00417161
15763.4	0.000531133	202654	15591.6	0.00364567
15769.2	-0.00657201	203868	15597.5	-0.00347261
15775.1	-0.00067204	203590	15603.3	0.00773537
15781	0.000754353	203280	15609.2	0.00527484
15786.9	-0.000215482	202874	15615.1	8.90E-05
15792.8	-0.00571025	202888	15621	-0.00272823
15798.6	-0.00249775	202568	15626.9	0.00228277
15804.5	-0.00618105	202310	15632.7	0.00289531
15810.4	0.00419742	201754	15638.6	-0.00231464
15816.3	0.0185135	199924	15644.5	-0.00246372
15822.2	0.00496486	201930	15650.4	-0.0066682
15828	0.0123571	201656	15656.3	-0.00109702
15833.9	0.0143548	201424	15662.2	-0.00561189
15839.8	0.0287937	201242	15668.1	0.00614525
15840.1	0.0350835	201194	15673.9	-0.00276673
15840.4	0.0262433	201166	15679.8	-0.00166585

15840.7	0.033519	201170	15685.7	0.000359505
15841	0.0321804	201088	15691.6	-0.00739965
15841.3	0.0334317	201086	15697.5	-0.00164293
15841.6	0.0298871	201090	15703.3	-0.00680217
15841.9	0.0324995	201092	15709.2	-0.0169073
15842.2	0.0307326	201040	15715.1	-0.00662214
15842.5	0.0346181	200996	15721	-0.00576578
15842.8	0.0351884	200944	15726.9	-0.00506288
15843.1	0.0360807	200944	15732.8	-0.00651774
15843.4	0.0325074	200962	15738.6	-0.00339055
15843.7	0.0343292	200912	15744.5	-0.00990206
15844	0.0324456	200846	15750.4	-0.00298591
15844.3	0.0351814	200840	15756.3	-0.00863513
15844.6	0.038761	200814	15762.2	-0.00378094
15844.9	0.041125	200828	15768	0.0193095
15845.2	0.0413241	200780	15773.9	0.000549167
15845.5	0.0390011	200726	15779.8	0.0081511
15845.8	0.0399841	200734	15785.7	-0.003674
15846.1	0.0426367	200696	15791.6	0.00188742
15846.4	0.0419536	200670	15797.5	0.0122413
15846.7	0.042659	200666	15803.4	0.0101687
15847	0.0459799	200592	15809.2	0.0138654
15847.3	0.0455923	200554	15815.1	0.00888503
15847.6	0.0450908	200542	15821	0.0100847
15847.9	0.0481306	200528	15826.9	0.0153131
15848.2	0.0518224	200492	15832.8	0.0210864
15848.5	0.0499187	200446	15838.6	0.0284977
15848.8	0.0478625	200386	15840.7	0.0337186
15849.1	0.0598929	200332	15841	0.031214
15849.4	0.0550663	200238	15841.3	0.0411536
15849.7	0.0590884	200084	15841.6	0.0386242
15850	0.0606455	200006	15841.9	0.0365497
15850.3	0.0574271	199868	15842.2	0.0351426
15850.6	0.0593213	199734	15842.5	0.0399344
15850.9	0.0618487	199676	15842.8	0.0361818
15851.2	0.0617253	199564	15843.1	0.0443016
15851.5	0.0647897	199436	15843.4	0.0397675
15851.8	0.065003	199324	15843.7	0.0358892
15852.1	0.0692603	199250	15844	0.033638
15852.4	0.0756715	199210	15844.3	0.0378372
15852.7	0.0712329	199088	15844.6	0.0395382
15853	0.0696652	198988	15844.9	0.0362235
15853.3	0.0793431	198902	15845.2	0.0419712
15853.6	0.0809613	198838	15845.5	0.0335645
15853.9	0.0766655	198862	15845.8	0.0412962

15854.2	0.0850523	198970	15846.1	0.0444032
15854.5	0.0864692	199044	15846.4	0.0424626
15854.8	0.0836983	199142	15846.7	0.0466488
15855.1	0.0874202	199344	15847	0.0466094
15855.4	0.0815718	199528	15847.3	0.0463275
15855.7	0.0940168	199612	15847.6	0.0435886
15856	0.0897654	199724	15847.9	0.0466073
15856.3	0.0969161	199916	15848.2	0.0484148
15856.6	0.0943065	200006	15848.5	0.0505855
15856.9	0.102414	199908	15848.8	0.0514754
15857.2	0.0970226	199964	15849.1	0.0526512
15857.5	0.109499	199984	15849.4	0.0573768
15857.8	0.108976	199944	15849.7	0.0599668
15858.1	0.108153	199936	15850	0.0508181
15858.4	0.111153	199888	15850.3	0.060041
15858.7	0.114784	199870	15850.6	0.0566239
15859	0.122016	199782	15850.9	0.0583246
15859.3	0.128439	199820	15851.2	0.0631622
15859.6	0.129944	199736	15851.5	0.0629153
15859.9	0.131463	199790	15851.8	0.0659492
15860.2	0.138984	199718	15852.1	0.0684281
15860.5	0.14614	199694	15852.4	0.0723319
15860.8	0.148516	199702	15852.7	0.0667279
15861.1	0.149521	199674	15853	0.0753973
15861.4	0.158366	199588	15853.3	0.0759678
15861.7	0.169973	199608	15853.6	0.0731349
15862	0.172662	199554	15853.9	0.0708082
15862.3	0.17343	199594	15854.2	0.0769522
15862.6	0.186281	199564	15854.5	0.0746202
15862.9	0.196255	199506	15854.8	0.0758578
15863.2	0.197273	199492	15855.1	0.0838833
15863.5	0.209632	199454	15855.4	0.0767696
15863.8	0.214464	199486	15855.7	0.0839277
15864.1	0.217405	199432	15856	0.0862966
15864.4	0.234542	199390	15856.3	0.0869713
15864.7	0.250538	199312	15856.6	0.0899408
15865	0.25726	199348	15856.9	0.0994507
15865.3	0.270812	199314	15857.2	0.105502
15865.6	0.288924	199284	15857.5	0.0999928
15865.9	0.293817	199276	15857.8	0.0998267
15866.2	0.31064	199214	15858.1	0.107856
15866.5	0.324065	199232	15858.4	0.111466
15866.8	0.348751	199170	15858.7	0.114771
15867.1	0.361136	199152	15859	0.116941
15867.4	0.391885	199152	15859.3	0.118639

15867.7	0.407793	199090	15859.6	0.125643
15868	0.427255	199064	15859.9	0.13425
15868.3	0.450301	199082	15860.2	0.128933
15868.6	0.485627	199002	15860.5	0.13758
15868.9	0.506693	198920	15860.8	0.138889
15869.2	0.548174	198982	15861.1	0.139202
15869.5	0.578533	198946	15861.4	0.151962
15869.8	0.61914	198932	15861.7	0.149991
15870.1	0.662882	198898	15862	0.160156
15870.4	0.712972	198906	15862.3	0.165042
15870.7	0.75748	198880	15862.6	0.162576
15871	0.818098	198820	15862.9	0.173553
15871.3	0.874722	198838	15863.2	0.179837
15871.6	0.93894	198728	15863.5	0.191026
15871.9	1.0059	198728	15863.8	0.201291
15872.2	1.09132	198708	15864.1	0.212892
15872.5	1.17238	198670	15864.4	0.218812
15872.8	1.26054	198628	15864.7	0.225603
15873.1	1.34927	198656	15865	0.242186
15873.4	1.44096	198600	15865.3	0.247344
15873.7	1.52676	198542	15865.6	0.253579
15874	1.61683	198526	15865.9	0.257759
15874.3	1.71182	198504	15866.2	0.282347
15874.6	1.80512	198490	15866.5	0.297759
15874.9	1.87822	198392	15866.8	0.311204
15875.2	1.95403	198398	15867.1	0.32579
15875.5	2.00499	198386	15867.4	0.332922
15875.8	2.05088	198366	15867.7	0.364615
15876.1	2.08187	198336	15868	0.373902
15876.4	2.1019	198234	15868.3	0.391167
15876.7	2.09586	198272	15868.6	0.418442
15877	2.07775	198192	15868.9	0.444014
15877.3	2.05274	198174	15869.2	0.468272
15877.6	2.01335	198124	15869.5	0.505663
15877.9	1.96665	198116	15869.8	0.534643
15878.2	1.91185	198038	15870.1	0.563189
15878.5	1.84761	197994	15870.4	0.600309
15878.8	1.78792	198004	15870.7	0.644693
15879.1	1.72418	197870	15871	0.693016
15879.4	1.65773	197818	15871.3	0.738124
15879.7	1.59431	197778	15871.6	0.799906
15880	1.53307	197786	15871.9	0.856546
15880.3	1.48629	197634	15872.2	0.911189
15880.6	1.42757	197602	15872.5	0.979106
15880.9	1.37777	197476	15872.8	1.04646

15881.2	1.34234	197438	15873.1	1.12206
15881.5	1.29955	197324	15873.4	1.20635
15881.8	1.26978	197158	15873.7	1.28292
15882.1	1.23355	197008	15874	1.37237
15882.4	1.20357	196802	15874.3	1.46361
15882.7	1.17662	196488	15874.6	1.54199
15883	1.14998	196152	15874.9	1.61979
15883.3	1.12899	195606	15875.2	1.69307
15883.6	1.11435	194858	15875.5	1.7657
15883.9	1.09275	194064	15875.8	1.82726
15884.2	1.08481	193148	15876.1	1.87019
15884.5	1.07244	192136	15876.4	1.91128
15884.8	1.06675	191022	15876.7	1.93892
15885.1	1.0573	190126	15877	1.96516
15885.4	1.048	189308	15877.3	1.96008
15885.7	1.04164	188612	15877.6	1.9598
15886	1.03991	188142	15877.9	1.94307
15886.3	1.03169	187886	15878.2	1.91039
15886.6	1.02195	187944	15878.5	1.87986
15886.9	1.0116	188204	15878.8	1.83805
15887.2	0.998433	189056	15879.1	1.79247
15887.5	0.989153	190630	15879.4	1.73372
15887.8	0.979115	192342	15879.7	1.68352
15888.1	0.961065	193654	15880	1.62271
15888.4	0.945955	194824	15880.3	1.57288
15888.7	0.937088	196060	15880.6	1.51181
15889	0.931649	197104	15880.9	1.46134
15889.3	0.917008	198060	15881.2	1.42371
15889.6	0.912249	198862	15881.5	1.37603
15889.9	0.899681	199452	15881.7	1.35138
15890.2	0.897366	199912	15882.1	1.30016
15890.5	0.889919	200106	15882.7	1.22999
15890.8	0.882192	200178	15883.4	1.16411
15891.1	0.883125	200194	15884	1.10893
15891.4	0.881972	200198	15884.7	1.06424
15891.7	0.880456	200162	15885.4	1.03292
15892	0.887905	200138	15886.2	0.99853
15892.3	0.872148	200128	15886.9	0.973058
15892.6	0.883207	200044	15887.7	0.950072
15892.9	0.883671	200040	15888.5	0.935009
15893.2	0.887337	200042	15889.3	0.917605
15893.5	0.882274	200050	15890.1	0.915994
15893.8	0.890446	199956	15891	0.905829
15894.1	0.887931	199966	15891.9	0.900889
15894.4	0.887852	199950	15892.7	0.903697

15894.7	0.899574	199870	15893.6	0.903696
15895	0.901476	199866	15894.6	0.919036
15895.3	0.905485	199814	15895.5	0.927948
15895.6	0.907196	199778	15896.5	0.928003
15895.9	0.912159	199752	15897.4	0.930869
15896.2	0.921233	199696	15898.5	0.940468
15896.5	0.922106	199690	15899.5	0.959485
15896.8	0.930743	199684	15900.5	0.994897
15897.1	0.934266	199610	15901.6	1.0297
15897.4	0.944674	199590	15902.6	1.05608
15897.7	0.948485	199594	15903.7	1.06534
15898	0.954513	199598	15904.9	1.07977
15898.3	0.968139	199524	15906	1.09755
15898.6	0.978476	199520	15907.1	1.10955
15898.9	0.985823	199506	15908.3	1.10649
15899.2	0.989999	199454	15909.5	1.10575
15899.5	0.997413	199486	15910.7	1.08611
15899.8	1.00819	199430	15911.9	1.08008
15900	1.02317	199394	15913.2	1.05921
15901.1	1.0466	199345	15914.4	1.04272
15902.2	1.07467	199536	15915.7	1.02506
15903.3	1.1045	199742	15917	1.0138
15904.4	1.12439	200114	15918.4	0.999323
15905.5	1.13675	200613	15919.7	0.985117
15906.7	1.13859	201231	15921.1	0.967585
15907.9	1.13482	201956	15922.4	0.954466
15909.1	1.12563	202820	15923.9	0.952153
15910.3	1.11855	203822	15925.3	0.948975
15911.6	1.09978	204865	15926.7	0.945648
15912.8	1.07692	206026	15928.2	0.934617
15914.1	1.05128	207387	15929.6	0.931433
15915.4	1.03834	208840	15931.1	0.927763
15916.7	1.01908	210376	15932.6	0.939202
15918	0.999706	212044	15934.2	0.941291
15919.4	0.988257	213906	15935.7	0.949204
15920.7	0.970612	215817	15937.3	0.961592
15922.1	0.95445	217882	15938.9	0.966364
15923.5	0.954774	220019	15940.5	0.985733
15925	0.942822	222292	15942.1	0.997706
15926.4	0.939009	224664	15943.8	1.00843
15927.9	0.934146	227265	15945.5	1.01658
15929.3	0.936816	229889	15947.1	1.02318
15930.8	0.93321	232656	15948.8	1.02728
15932.4	0.940598	235502	15950.6	1.0356
15933.9	0.955377	238463	15952.3	1.03739

15935.5	0.960437	241601	15954.1	1.03613
15937.1	0.971797	244853	15955.8	1.03764
15938.7	0.983884	248118	15957.7	1.03353
15940.3	0.998577	251568	15959.5	1.03415
15941.9	1.00833	255209	15961.3	1.02527
15943.6	1.01439	258879	15963.2	1.0158
15945.2	1.02483	262684	15965	1.01318
15946.9	1.03495	266559	15966.9	1.00993
15948.7	1.04445	270586	15968.9	1.00294
15950.4	1.0352	274798	15970.8	0.993078
15952.1	1.04624	279099	15972.7	0.993263
15953.9	1.049	283456	15974.7	0.987686
15955.7	1.04599	287978	15976.7	0.981822
15957.5	1.04829	292605	15978.7	0.975102
15959.3	1.0449	297281	15980.7	0.973733
15961.2	1.0421	301949	15982.8	0.97427
15963	1.03799	305968	15984.9	0.970731
15964.9	1.02878	311775	15986.9	0.976403
15966.8	1.02359	317455	15989	0.984394
15968.7	1.01885	322732	15991.2	0.982339
15970.7	1.00863	328179	15993.3	0.987827
15972.6	1.00633	333715	15995.5	0.996673
15974.6	0.99578	339356	15997.7	0.996104
15976.6	0.99145	345112	15999.9	1.00035
15978.6	0.980987	350921	16002.1	1.005
15980.7	0.976444	356907	16004.3	1.0071
15982.7	0.97627	362939	16006.6	1.00538
15984.8	0.973372	369199	16008.9	1.0069
15986.9	0.981889	375521	16011.1	1.0127
15989	0.984457	381820	16013.5	1.00671
15991.1	0.989228	388402	16015.8	1.00127
15993.3	0.993245	395019	16018.1	1.00151
15995.5	0.994121	401816	16020.5	0.999041
15997.7	1.00016	408744	16022.9	1.00059
15999.9	1.00669	415729	16025.3	0.996459
16002.1	1.00809	422753	16027.7	0.993259
16004.3	1.01109	429959	16030.2	0.99345
16006.6	1.01954	436094	16032.7	0.995667
16008.9	1.01839	444401	16035.1	0.987291
16011.2	1.02085	452320	16037.6	0.985078
16013.5	1.0184	459983	16040.2	0.985975
16015.8	1.01442	467782	16042.7	0.989682
16018.2	1.01205	475408	16045.3	0.989939
16020.6	1.01474	483459	16047.9	0.987285
16023	1.00928	491501	16050.5	0.993056

16025.4	1.00923	499580	16053.1	0.989613
16027.8	1.00857	507847	16055.7	0.987951
16030.3	1.00252	516242	16058.4	0.991828
16032.8	1.00454	524132	16061.1	0.996611
16035.3	1.00456	533323	16063.8	0.99482
16037.8	0.99565	542171	16066.5	0.994814
16040.3	0.99818	550950	16069.2	0.99188
16042.9	0.998231	559517	16072	0.997017
16045.4	0.996878	568192	16074.7	0.99715
16048	0.993932	578139	16077.5	0.998034
16050.6	0.993524	587350	16080.3	1.0015
16053.3	0.996089	596736	16083.2	0.998354
16055.9	1.00118	606175	16086	0.998911
16058.6	0.998707	615695	16088.9	0.997678
16061.3	1.00063	625360	16091.8	1.00072
16064	1.00061	634788	16094.7	0.999236
16066.7	0.997434	644817	16097.6	1.00091
16069.4	1.00083	654903	16100.5	0.999155
16072.2	1.00111	669222	16103.5	0.996797
16075	1.00248	682433	16106.5	0.997634
16077.8	1.00656	692456	16109.5	0.995696
16080.6	1.0057	703384	16112.5	0.995925
16083.4	1.00498	713835	16115.5	1.00352
16086.3	1.00533	724400	16118.6	1.01871
16089.2	1.0077	735368	16121.7	0.998755
16092	1.00679	746330	16124.8	0.999906
16094.9	1.01031	757180	16127.9	0.995928
16097.9	1.01178	768279	16131	0.99571
16100.8	1.01229	779042	16134.2	0.995803
16103.8	1.01014	789097	16137.3	0.998014
16106.8	1.00371	802019	16140.5	0.999868
16109.8	1.00124	813379	16143.7	1.00034
16112.8	0.999656	824959	16147	1.00499
16115.9	0.997431	836247	16150.2	1.01665
16118.9	0.997675	847512	16153.5	0.99548
16122	1.00045	857936	16156.8	0.998428
16131.4	0.994583	856519	16160.1	0.995602
16134.6	0.994224	841321	16163.4	0.998342
16144.1	0.99613	894717	16166.7	1.00013
16153.9	0.999497	908767	16170.1	0.996105
16157.2	0.992884	920992	16173.5	0.999655
16160.5	0.99502	934555	16176.9	0.995148
16163.8	0.996421	946088	16180.3	0.996409
16167.2	1.0009	948718	16183.7	1.00065
16170.6	0.99679	966179	16187.2	1.00018

16184.2	0.996118	984040	16190.7	1.00108
16187.7	0.993393	997186	16194.2	0.998615
16191.2	0.998547	1.01E+06	16197.7	0.999816
16194.7	0.999475	1.02E+06	16201.2	1.0023
16198.2	0.998876	1.04E+06	16204.8	1.00423
16201.7	0.999219	1.05E+06	16208.4	1.00173
16205.3	0.997731	1.06E+06	16211.9	0.999884
16208.9	0.995475	1.08E+06	16215.5	1.00107
16212.5	0.996489	1.09E+06	16219.2	0.998311
16216.1	0.997963	1.10E+06	16222.8	1.00471
16219.8	1.00042	1.12E+06	16226.5	1.00279
16223.4	1.00014	1.13E+06	16230.2	1.00057
16227.1	0.999533	1.14E+06	16233.9	1.00347
16230.8	0.997029	1.16E+06	16237.6	0.997857
16234.5	0.996075	1.17E+06	16241.4	1.0007
16238.2	0.998146	1.18E+06	16245.1	0.999629
16242	1.00017	1.20E+06	16248.9	1.00587
16245.7	0.998827	1.21E+06	16252.7	1.00203
16249.5	0.99808	1.23E+06	16256.5	1.00042
16253.3	0.999444	1.24E+06	16260.4	1.00722
16257.2	0.998332	1.26E+06	16264.2	1.00386
16261	0.999272	1.27E+06	16268.1	1.00564
16264.9	0.998749	1.29E+06	16272	1.00387
16268.7	0.999632	1.30E+06	16275.9	1.00518
16272.7	0.996968	1.33E+06	16279.8	1.00101
16276.6	1.00053	1.34E+06	16283.8	1.00667
16280.5	0.998457	1.36E+06	16287.7	1.00424
16284.5	0.996801	1.37E+06	16291.7	1.00756
16288.5	0.997507	1.39E+06	16295.8	1.00633
16292.5	0.997463	1.40E+06	16299.8	1.00909
16296.5	0.997712	1.42E+06	16303.8	1.00611
16300.5	0.99686	1.43E+06	16307.9	1.00368
16304.6	0.992447	1.45E+06	16312	1.00598
16308.6	0.998587	1.47E+06	16316.1	1.00318
16312.7	0.998862	1.48E+06	16320.2	1.00095
16316.9	0.998802	1.50E+06	16324.4	1.00121
16321	1.00034	1.51E+06	16328.5	1.00239
16325.1	1.00151	1.53E+06	16332.7	1.00086
16329.3	1.00123	1.55E+06	16336.9	1.00345
16333.5	1.00215	1.56E+06	16341.1	1.0046
16337.7	1.00189	1.58E+06	16345.4	0.999551
16341.9	1.00335	1.59E+06	16349.6	1.00329
16346.2	1.00144	1.61E+06	16353.9	0.999074
16350.4	1.00147	1.63E+06	16358.2	1.00044
16354.7	0.996822	1.64E+06	16362.5	1.00366

16359	1.00141	1.66E+06	16366.8	1.00135
16363.3	1.00236	1.68E+06	16371.2	1.00034
16367.7	1.00094	1.69E+06	16375.6	1.0035
16372.1	0.998171	1.71E+06	16379.9	1.00164
16376.4	1.0019	1.72E+06	16384.4	0.998817
16380.8	0.999633	1.74E+06	16388.8	1.00204
16385.3	1.00051	1.76E+06	16393.2	1.0024
16389.7	1.00382	1.77E+06	16397.7	1.00118
16394.1	0.999975	1.79E+06	16402.2	1.00038
16398.6	1.00012	1.81E+06	16406.7	1.00316
16403.1	1.00305	1.82E+06	16411.2	1.00375
16407.6	0.999617	1.86E+06	16415.8	1.00346
16412.1	1.00018	1.88E+06	16420.3	1.00103
16416.7	0.998219	1.90E+06	16424.9	0.999073
16421.3	1.00256	1.91E+06	16429.5	0.998155
16425.9	1.00246	1.93E+06	16434.1	0.999339
16430.5	0.999987	1.95E+06	16438.8	0.996367
16435.1	0.997772	1.97E+06	16443.4	0.996651
16439.7	1.00231	1.98E+06	16448.1	1.00094
16444.4	0.998366	2.00E+06	16452.8	0.995714
16449.1	0.999934	2.02E+06	16457.5	0.998956
16453.8	0.998313	2.04E+06	16462.3	0.996541
16458.5	0.9984	2.06E+06	16467	0.997164
16463.3	1.00084	2.07E+06	16471.8	0.994061
16468	0.997955	2.09E+06	16476.6	0.993671
16477.6	0.998493	2.11E+06	16481.4	0.998907
16482.4	1	2.13E+06	16486.2	0.993523
16487.2	0.999043	2.15E+06	16491	0.995576
16497	1.00292	2.17E+06	16495.9	0.997675
16501.9	1.00102	2.18E+06	16500.8	0.998642
16506.8	1.00153	2.20E+06	16505.7	0.996099
16511.7	1.00113	2.22E+06	16510.6	0.998693
16516.7	0.997742	2.24E+06	16515.6	0.996312
16521.6	1.00033	2.26E+06	16520.5	1.00005
16526.6	0.998714	2.28E+06	16525.5	0.998591
16531.6	1.00012	2.30E+06	16530.5	0.996874
16536.7	1.00772	2.34E+06	16535.5	1.00038
16541.7	0.996446	2.36E+06	16540.6	0.994464
